# Where to live? Landfast sea ice shapes emperor penguin habitat around Antarctica

**DOI:** 10.1126/sciadv.adg8340

**Published:** 2023-09-27

**Authors:** Sara Labrousse, David Nerini, Alexander D. Fraser, Leonardo Salas, Michael Sumner, Frederic Le Manach, Stephanie Jenouvrier, David Iles, Michelle LaRue

**Affiliations:** ^1^ Laboratoire d’Océanographie et du Climat: Expérimentations et approches numériques (LOCEAN), UMR 7159 Sorbonne-Université, CNRS, MNHN, IRD, IPSL, 75005 Paris, France.; ^2^ Mediterranean Institute of Oceanography, MIO, Aix-Marseille University, Marseille, France.; ^3^ Australian Antarctic Program Partnership, Institute for Marine and Antarctic Studies, University of Tasmania, Hobart, Tasmania.; ^4^ Point Blue Conservation Science, Petaluma, CA, USA.; ^5^ Integrated Digital East Antarctica, Australian Antarctic Division, Channel Highway, Kingston, Tasmania 7050, Australia.; ^6^ BLOOM, 16 rue Martel, 75010 Paris, France.; ^7^ Department of Biology, Woods Hole Oceanographic Institution, Woods Hole, MA, USA.; ^8^ Canadian Wildlife Service, Environment and Climate Change Canada, Ottawa, Canada.; ^9^ Department of Earth and Environmental Science, University of Minnesota, Minneapolis, MN, USA.; ^10^ School of Earth and Environment, University of Canterbury, Christchurch, New Zealand.

## Abstract

Predicting species survival in the face of climate change requires understanding the drivers that influence their distribution. Emperor penguins (*Aptenodytes forsteri*) incubate and rear chicks on landfast sea ice, whose extent, dynamics, and quality are expected to vary substantially due to climate change. Until recently, this species’ continent-wide observations were scarce, and knowledge on their distribution and habitat limited. Advances in satellite imagery now allow their observation and characterization of habitats across Antarctica at high resolution. Using circumpolar high-resolution satellite images, unique fast ice metrics, and geographic and biological factors, we identified diverse penguin habitats across the continent, with no significant difference between areas with penguins or not. There is a clear geographic partitioning of colonies with respect to their defining habitat characteristics, indicating possible behavioral plasticity among different metapopulations. This coincides with geographic structures found in previous genetic studies. Given projections of quasi-extinction for this species in 2100, this study provides essential information for conservation measures.

## Teaser

Emperor penguins do not have one but many Antarctic habitats, which could be key for their survival in the short term.

## INTRODUCTION

Human actions are causing increased extinction of species around the world that is tens to hundreds of times higher than the average rate over the past 10 million years (*1*). There is an urgent need for understanding the processes that construct and maintain species habitats, to determine potential future refugia for threatened species, bring attention to their fate, and determine efficient conservation measures to safeguard them. The emperor penguin (*Aptenodytes forsteri*) is an iconic Antarctic species that is threatened by climate change and associated sea ice losses over the past century (*2*, *3*), as their breeding habitat is critically dependent on seasonal sea ice (*4*). Hence, only a drastic reduction in anthropogenic greenhouse gas emissions would reduce threats for this species, and failing to do so could result in important declines in emperor penguin populations (*5*–*7*).

Since the inception of regular satellite monitoring in late 1978, Antarctic sea ice has shown an overall near-zero trend of surface extent, despite regionally contrasted variations and considerable interannual variability (*8*). Most climate models indicate that Antarctic sea ice extent should have decreased over the past several decades (*9*). However, multiple anthropogenic forcings (ozone and greenhouse gases) and complicated processes involving the ocean, atmosphere, and adjacent ice sheet are leading to low confidence in projections of Antarctic sea ice (*10*). As a result, models cannot be used to characterize the sea icescape at high spatial resolution, as they for instance provide crude estimates of landfast ice, thereby hampering habitat modeling of sea ice–dependent species. Given that emperor penguins are sensitive to local and regionally contrasted sea ice conditions on the short to medium terms (*10*, *11*), and given the complexity of the main drivers of Antarctic sea ice and limits of climate models, threats to emperor penguin on the short-term associated with sea ice changes are uncertain and difficult to predict from one region to another. Therefore, we need to understand the diversity of habitats and fine scale parameters that shape emperor penguin presence in Antarctica.

Emperor penguins are, throughout their breeding period, intricately linked to landfast ice (henceforth, “fast ice”), i.e., the narrow band of coastal, compact sea ice that is held in place by ice shelves and grounded icebergs (*12*). Unfortunately, measuring and modeling fast ice remains challenging at the circumpolar scale (*13*, *14*), and trends in coastal fast ice may be independent of those in sea ice extent (*2*). Loss of fast ice such as early breakout can cause massive breeding failure and even adult mortality in emperor penguins (*5*, *15*). The importance and medium-term impact of occasional massive perturbations are only now becoming apparent (*16*, *17*).

We sought to describe the habitat of emperor penguins through an approach that helps identify the underlying processes constructing and maintaining those habitats. Here, we first investigated the diversity of habitats shaping the presence of emperor penguin around Antarctica based on unique fast ice extent and variability data, intra/interspecific trophic competition factors, and geography.

The first part of our study was made possible by the recent publication of a new, unique time series of fast ice extent from March 2000 to March 2018 (*18*), which contains 432 contiguous maps of fast ice extent at a 1 km and 15-day resolution. This dataset, created from NASA Moderate Resolution Imaging Spectroradiometer (MODIS) sensors onboard the Terra and Aqua satellites (*19*, *20*) is revolutionizing the way emperor penguin habitat can be characterized. We assumed that changes in fast ice persistence, seasonality, timing of the maximum and minimum extents, and frequency of breaking out would influence where the emperor penguins would settle their breeding colonies. We also assumed that intraspecific competition may determine the distribution of emperor penguin colonies around Antarctica [colonies are evenly spaced ∼220 km apart; (*21*)]. Similarly, we hypothesized that interspecific trophic competition [trophic competitors are Adélie penguins (*Pygoscelils adeliae*) and Weddell seals (*Leptonychotes weddellii*)] may determine the geography locations of emperor penguin colonies (*21*, *22*). Last, geography such as the slope of the bathymetry, distance to certain isobaths, and ocean depths may aggregate prey where local upwellings stimulate primary production (*23*). Previous work suggests that regions with particular bathymetric features over or near the continental slope were prime foraging habitat for emperor penguins during the breeding season (*24*–*29*). Thus, geography is an important parameter to consider regarding emperor penguin habitat around Antarctica.

Species distribution combined with interdisciplinary science (short-term sea ice dynamics, geographic, and trophic competition) are essential for understanding ecological responses and population viability to their environment widely, but also at a regional scale. Here, we assess the habitat-species relationships of emperor penguin and emphasize the importance of considering metapopulations with hypothesis on their behavioral plasticity and dispersal abilities as adaptive tools against habitat change locally and at regional scales, which are of important consequences for management and conservation on decadal time frames.

## RESULTS

**Fig. 1.**
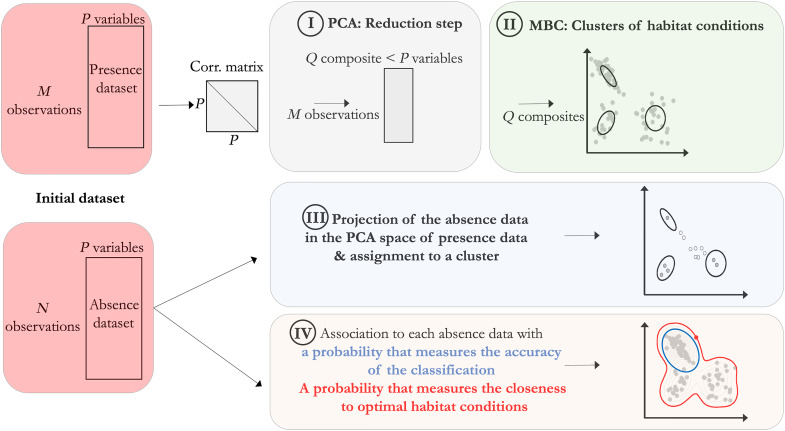
Workflow of the design of the study composed of four main steps. A PC analysis is achieved on the correlation matrix of presence data (step I). Using four PCs of this analysis, parameters of the model-based clustering are estimated and presence data are clustered in five groups (step II). Absence observations are then projected in the presence’s PC analysis space and assigned to a single cluster given with model-based clustering (step III). Using the properties of the mixture Gaussian model provided by the model-based clustering, a probability that measures the accuracy of the classification can be associated to each absence data and a probability that measures the closeness to optimal habitat conditions (step IV).

**Fig. 2.**
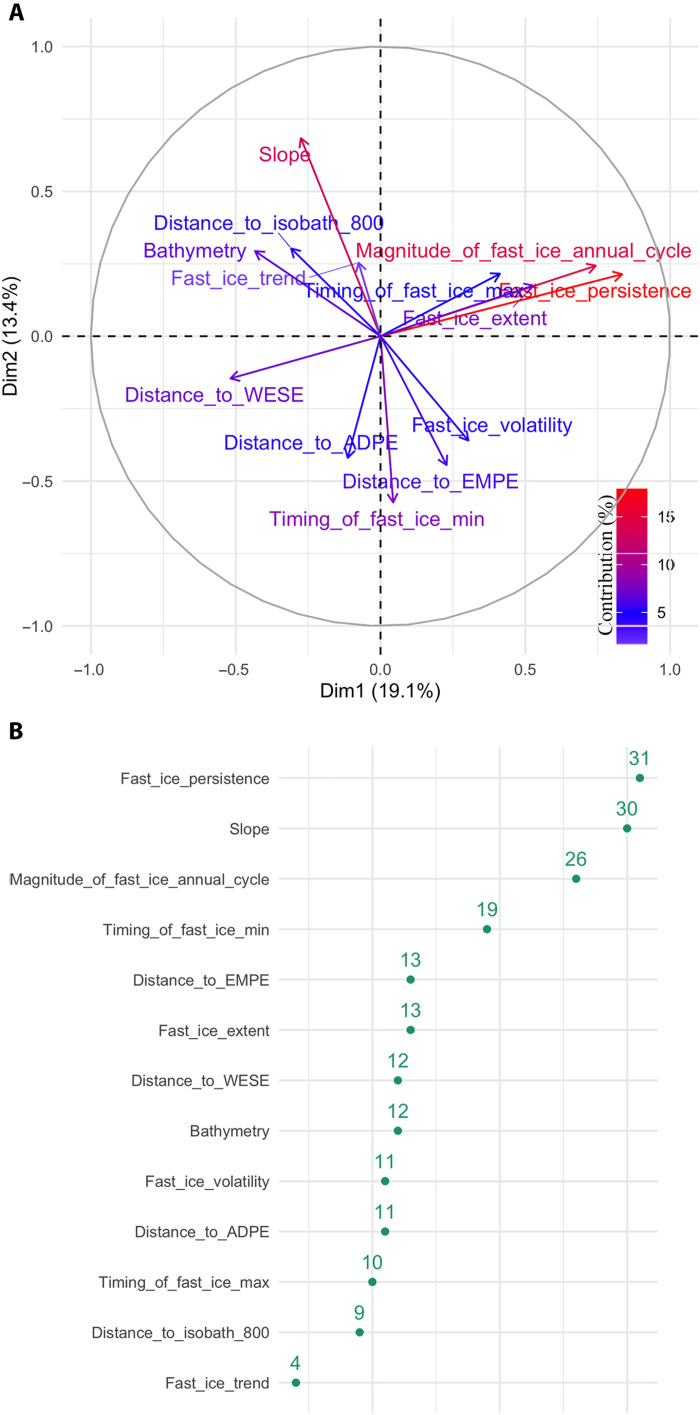
PC analysis. Representation of the projection of the variables into the two-dimension space for the emperor penguin presence data. (**A**) represents the first and second components and (**B**) the variable contribution to variance considering the first two components. The first PC axis explained 19% of variance in habitat conditions across occupied colonies and loaded heavily on fast ice variables: fast ice persistence (contribution of 28%; cos^2^ of 0.7) and the magnitude of the fast ice annual cycle (contribution of 22%; cos^2^ of 0.55). Positive values on this axis describe colonies with greater fast ice persistence and greater amplitude in the magnitude of the fast ice annual cycle. The second PC axis explained an additional 13% of variance in habitat conditions and loaded heavily on the slope of the bathymetry (contribution of 27%; cos^2^ of 0.47). Positive values on this axis correspond to greater slope index.

The developed approach for the study of emperor penguin habitat relies on two main methods (see details in Methods and Discussion): principal component (PC) analysis (steps 1 and 3 of Fig. 1) of the environmental variables and a Bayesian statistical approach for classification (model-based clustering, steps 2 and 3 of Fig. 1). PC analysis on the 177 presence data was conducted on 13 input variables (three geographic, seven fast ice, and three biological variables; step 1 in Fig. 1). The analysis revealed that physical variables, including fast ice persistence, its magnitude, and the slope of the bathymetry best describe variation in emperor penguin habitat, accounting for 32% of variability. The first PC axis explained 19% of variance in habitat conditions across occupied colonies, and loaded heavily on fast ice variables: fast ice persistence (contribution of 28%; cos^2^ of 0.7; Fig. 2) and the magnitude of the fast ice annual cycle (contribution of 22%; cos^2^ of 0.55; Fig. 2). Positive values on this axis describe colonies with greater fast ice persistence and greater amplitude in the magnitude of the fast ice annual cycle. The second PC axis explained an additional 13% of variance in habitat conditions and loaded heavily on the slope of the bathymetry (contribution of 27%; cos^2^ of 0.47; Fig. 2). Positive values on this axis correspond to greater slope index. The third PC explained an additional 11.08% (fig. S1) and was represented by the distance to Adélie penguin colonies (contribution of 36%; cos^2^ of 0.51) and the distance to isobath 800 m (contribution of 32%; cos^2^ of 0.22). Positive values on this axis correspond with short distance to Adélie penguin colonies and long distances to the isobath 800 m.

Absence observations were then projected in the space of the previous PC analysis so as to summarize the 13 variables in composite PC coordinates (step 3 in Fig. 1). Displaying the first three PC coordinates of colony locations into geographical space allows visualizing spatial patterns in conditions that are consistent with the presence of emperor penguin colonies. Some spatial structure arises and are described in fig. S2.

**Fig. 3.**
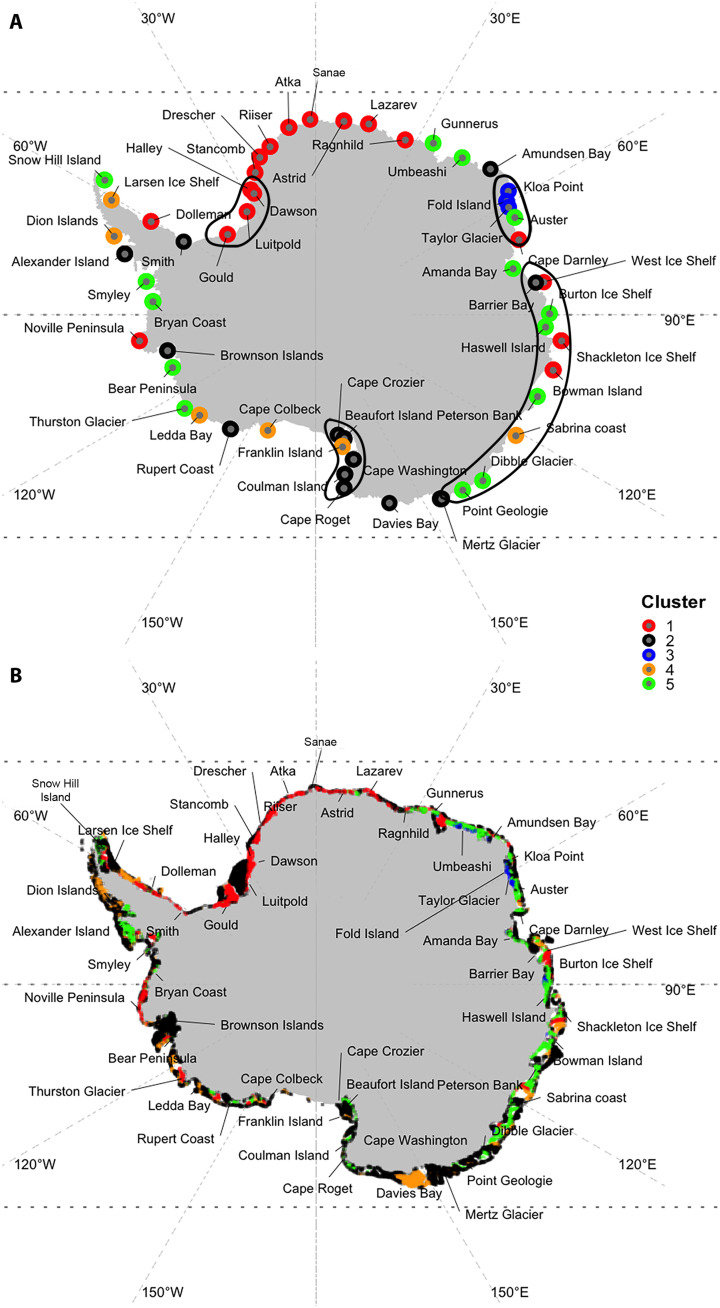
Antarctic maps with habitat clusters displayed for each emperor penguin colony. (**A**) Hard clustering obtained with the model-based clustering on the presence data. (**B**) Hard clustering of absence data conditionally to model-based clustering on presence data. For (A), all colonies are assigned to a single cluster. For (B), only data with 80% certainty in the classification are represented. Four geographical regions were observed: (i) the region from Gould (∼47°W) to Ragnhild (∼27°E), named the Weddell Sea region, was dominated by the first cluster; then, (ii) the region from Gunnerus (∼34°E) to Dibble Glacier (∼135°E), the East Antarctica region, was dominated by cluster 5 with the exception of Kloa Point, Fold Island, and Taylor Glacier (∼60°E); (iii) in the region from Pointe Geologie (∼140°E) to Cape Crozier (∼169°E), the Ross Sea region, colonies were associated with cluster 2; and last, (iv) in the region from Cape Colbeck (∼157°W) to Smith (∼60°W), the West Antarctic region, we found a mix of clusters 2, 4, and 5. Black polygons on (A) refer to the four genetic metapopulations identified in (*30*).

**Fig. 4.**
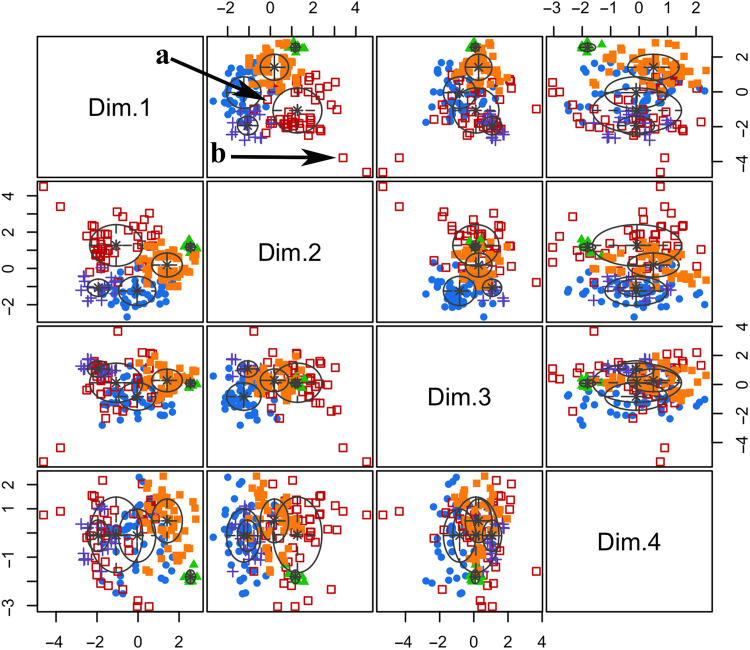
Model-based clustering display based on the four PC. Each color corresponds with one of the five identified clusters. The arrow with the letter “a” represents for one observation with a high probability to be close to the center of the five clusters (i.e., probability to be close to high-density habitat areas) but a low accuracy to belong to the red cluster. In contrast, the arrow with the letter “b” corresponds with a high accuracy to belong to the red cluster but a low probability to be close to high-density habitat areas.

Colonies were then clustered into a small number of classes that share similar environmental conditions by running a model-based clustering (step 2 in Fig. 1) analysis using coordinates of the first four PC (corresponding to 55% of the cumulated variance; fig. S1). The model proposed five clusters (log likelihood of −1109, Bayesian Information Criterion (BIC) of 2384, Integrated Complete Likelihood (ICL) of −2430, *n* = 177, and df = 32) composed of 49, 47, 8, 20, and 53 emperor penguin presence observations, respectively. Taking an arbitrary threshold of 80% certainty of cluster attribution, 50 colonies of 55 were assigned a cluster (Figs. 3A and 4), and among those 50, three colonies (Alexander Island, Bowman Island, and Dresher) can be assigned to two clusters. The five colonies without cluster based on the 80% threshold were West Ice Shelf, Barrier Bay, Thurston Glacier, Cape Colbeck, and Cape Washington. When using a threshold of 59% certainty, all the 55 colonies had an identified cluster. On the basis of the 80% threshold, clusters 1 through 5 had 15, 16, 3, 6, and 13 colonies, respectively.

To identify regions sharing consistent environmental conditions, we then predicted those clusters on the absence data when projecting observations in the presence’s PC analysis space (step 3 in Fig. 1). The geographic structure of these clusters for a given threshold above 80% is very similar to the one described for Fig. 3A (Fig. 3B).

**Fig. 5.**
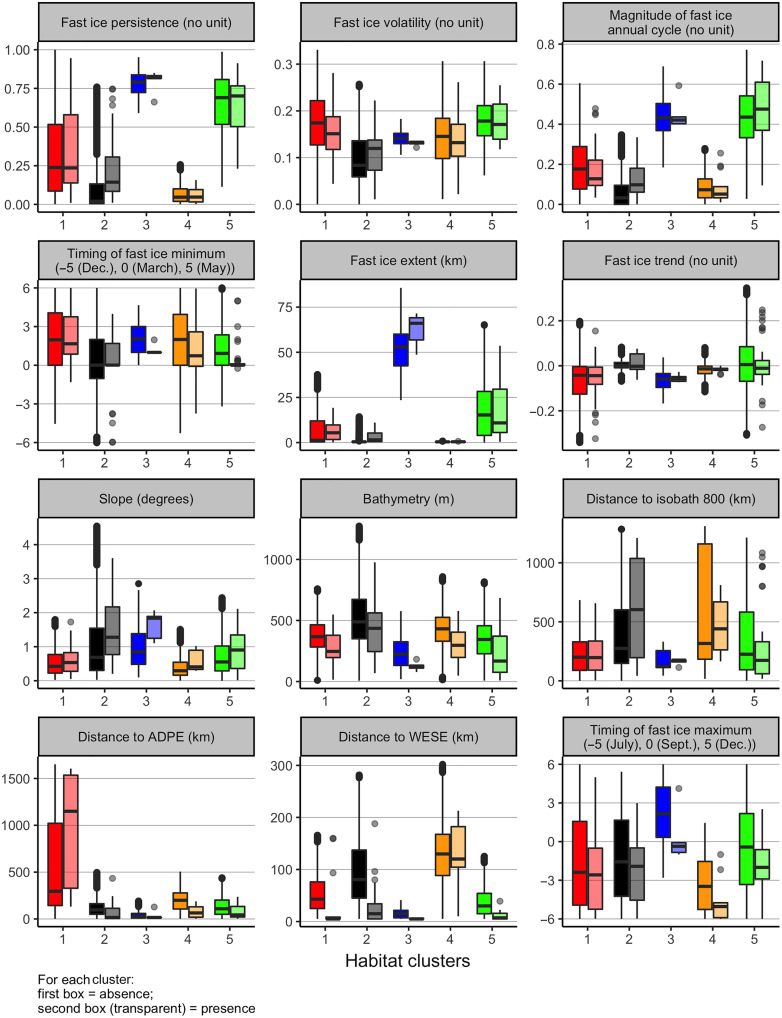
Boxplots of each habitat cluster for each variable and for presence (lighter color) and absence (darker color) data. Only data with 80% certainty in the classification are represented. For illustration purpose, we removed the variable “distance to the nearest emperor penguin colony” as this distance is very similar among regions and between colonies as detailed in the Results and Discussion. For each cluster, presence data are not significantly different from absence data for the most contributing variables. Variables that explained habitat for emperor penguins in the different regions are described in Tables 1 and 2.

Last, to understand emperor penguin habitat requirements, we then represented for each cluster the range of value for each variables for both presence and absence based on a threshold of 80% certainty (Fig. 5). All medians and SDs are available in table S1. Significance at 5% level between clusters for each variable is available in fig. S3 and table S2. For each cluster, presence data are not significantly different from absence data for the most contributing variables (fig. S4 and table S3). We removed absence data within some range (10, 20, and 30 km) around each colonies and tested again the significance between absence and presence data for each cluster. The significance did not change [except for 30 km, one cluster (*5*) for only one variable (fast ice persistence); tables S4 to S6].

We observed four geographical regions: (i) the region from Gould (∼47°W) to Ragnhild (∼27°E), named the Weddell sea region, was dominated by the first cluster; then (ii) the region from Gunnerus (∼34°E) to Dibble Glacier (∼135°E), the East Antarctica region, was dominated by cluster 5 with the exception of Kloa Point, Fold Island, and Taylor Glacier (∼60°E); (iii) in the region from Pointe Géologie (∼140°E) to Cape Crozier (∼169°E), the Ross Sea region, colonies were associated with cluster 2; and last, (iv) in the region from Cape Colbeck (∼157°W) to Smith (∼60°W), the West Antarctic region, we found a mix of clusters 2, 4, and 5 (Table 1 and Fig. 3A). Variables that explained habitat for emperor penguins in the different regions are described in Tables 1 and 2.

**Table 1.** Description of each habitat cluster including the geographic extent and the main environmental characteristics.

Habitat cluster	Geographic extent	Environmental characteristics
1	Weddell Sea	Medium fast ice persistence
		Medium volatility
		Low fast ice extent
		Low magnitude of the annual cycle of fast ice growth and retreat
		Negative fast ice trend
5	East Antarctica	High fast ice persistence
		High amplitude of the annual cycle
		No fast ice trend
		Close distance to Adelie penguin colonies and Weddell seal colonies
2	Ross sea	Low fast ice persistence
		Low magnitude of the annual cycle
		Close distance to Adelie penguin colonies
		Far distance from Weddell seals
3	3 colonies (Kloa Point, Fold Island, and Taylor Glacier)	High fast ice extent
		Late fast ice maximum (December)
Several classes incl. cluster 4	West Antarctica	Lowest fast ice persistence (class 4)
		Low magnitude of the annual cycle (class 4)
		No fast ice trend (class 4)
		Low fast ice extent (class 4)

**Table 2.** Description of the median values (±SD) for the main fast ice variables representing each habitat cluster and associated regions.

Regions	Fast ice persistence (0–100%)		Fast ice volatility (0–1)		Magnitude of the fast ice annual cycle (0–1)		Fast ice extent (km)		Fast ice trends	
	Presence	Absence	Presence	Absence	Presence	Absence	Presence	Absence	Presence	Absence
Cluster 1/Weddell Sea	24 ± 31%	24 ± 29%	0.15 ± 0.06	0.17 ± 0.07	0.13 ± 0.11	0.18 ± 0.14	6 ± 12 km	3 ± 16 km	−0.04 ± 0.11	−0.04 ± 0.13
Cluster 5/East Antarctic	24 ± 31%	70 ± 16%	0.17 ± 0.04	0.18 ± 0.04	0.48 ± 0.16	0.44 ± 0.15	11 ± 15 km	15 ± 16 km	−0.01 ± 0.1	−0.01 ± 0.14
Cluster 2/Ross Sea	20 ± 29%	3 ± 30%	-	-	0.17 ± 0.18	0.03 ± 014	4 ± 18 km	0.5 ± 17 km	-	-
Cluster 3/3 colonies	-	-	-	-	-	-	66 ± 9 km	53 ± 14 km	-	-

**Fig. 6.**
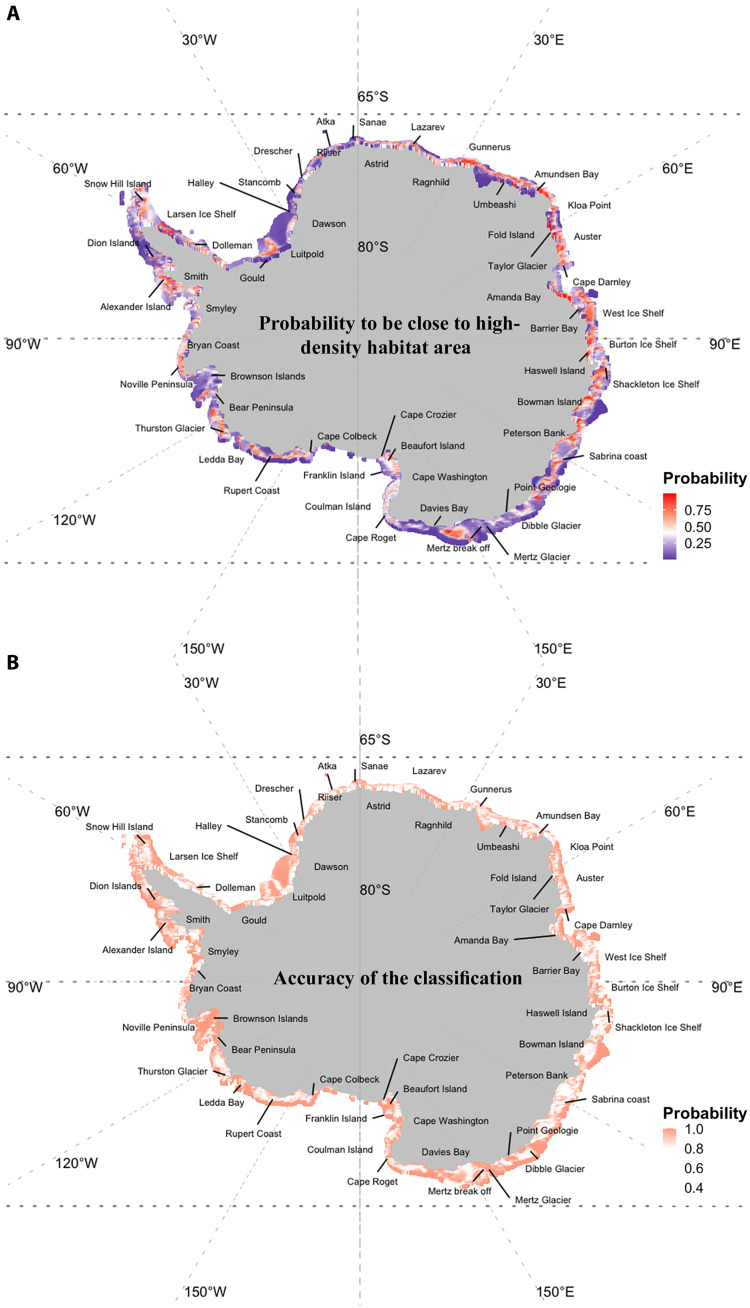
Model-based clustering quality. (**A**) represents the probability to be close to high-density habitat areas (i.e., probability to be close to the center of the five clusters) and (**B**) represents a measure of accuracy to belong to a given cluster. For example, the Weddell Sea has mainly observations with a low probability of being close to high-density areas (dark blue areas), while they have a high probability to be classified in a given cluster (red areas). In contrast, the East Antarctic, Ross Sea, and West Antarctic regions have many observations with a high probability of being close to high-density areas (red areas), while their certainty to belong to a given cluster is low in some particular pocket areas.

We then computed the probability to be close to high-density habitat areas (i.e., to be close to the center of the five clusters, examples in Figs. 4 and 6A) and conditional probability to belong to a given cluster (examples in Figs. 4 and 6B), which provide some complementary information on the hard clustering (step 4 in Fig. 1). We found that the Weddell Sea region, for example, has mainly observations with a low probability of being close to high-density areas (dark blue areas), while they have a high probability to be classified in a given cluster (red areas; Fig. 6, A and B). In contrast the East Antarctic, Ross Sea and West Antarctic regions have many observations with a high probability of being close to high-density areas (red areas), while their certainty to belong to a given cluster is low in some particular pocket areas (white areas; Fig. 6, A and B).

## DISCUSSION

Our study reveals that emperor penguins apparently use a range of different type of habitats, presumably, the habitat available to them when they were born. Crucially, we show that the different habitats were matching the four known genetic metapopulations (*30*). We did not find difference between habitats where emperor penguins are present or absent; however, our sample sizes may not be sufficient to correctly assess this difference. This study radically changes our perceptions of the “emperor penguin habitat,” which is generally described as a unique type of habitat and indicates behavioral plasticity among different metapopulations of penguins. On the basis of the apparent absence of differences between absence and presence data, we hypothesized that intraspecific competition may be one of the most important factor conditioning the distribution of emperor penguin colonies around Antarctica as colonies are evenly spaced (∼220 km apart) around Antarctica (*21*).

Previous studies have described the consequences of habitat variability on breeding success or population numbers of emperor penguins based on sea ice extent (*6*, *15*, *31*–*33*), fast ice extent (*13*, *34*, *35*), wind strength, air and sea temperatures, fast ice extent and polynyas (*36*), or distance to neighboring colonies, polynyas, and submarine canyons (*21*). The latter suggested that the gaps between colonies may well represent areas where emperor penguins could move to if current locations become unfavorable/unavailable; the research here would support that result, since there is no apparent difference between the variables explaining absence or presence of emperors. However, previous studies mostly concerned one or two colonies, presence-only data, and were based on large temporal or spatial scales. Other studies have highlighted the consequences on breeding success or population numbers of extreme event or early fast ice break up (*17*, *37*, *38*), and most studies project population numbers based on large-scale sea ice extent from climate models (*2*, *39*). Our study is the first to describe and quantify the circumpolar emperor penguin habitats at fine scale, which was previously hampered by the difficulty to characterize fast ice at fine scale and over long time periods.

Our methodology and results, based on a unique and unbalanced set of presence/absence data, constitute a useful tool for characterizing habitat-species relationships and for identifying potential refugia in the future among a set of environmental variables for both terrestrial and marine animals. This tool is transient and could be transferred and improved with the use of new variables and higher spatio-temporal resolution.

The developed approach relies on two main methods: PC analysis of the environmental variables (*40*) and a Bayesian statistical approach for classification [model-based clustering; (*41*)]. PC analysis is a widely used method especially for the analysis of environmental data (*40*). In our case, this method presents two main advantages. First, it is a dimension reduction technique, which allows summarizing the set of environmental variables into a small number of composite variables (the PCs). It is especially advised in our case because the number of presence observations is quite low regarding the number of variables. Reducing the dimension of variables gives more robustness to outlier observations (*42*), and is especially recommended before any classification purpose as the density of observations increases in a low-dimensional space. The second advantage of the PC analysis is its ability to easily project new observations sampled on every location in this reduced space of habitat. Absence data can be positioned conditionally to the structure of the PC space of habitat conditions built on presence data and then easily compared. In the same way, the inference step provided with the Bayesian approach used for clustering habitat conditions on presence data is also well suited when the number of observations is quite low. The construction of the mixture Gaussian model with some simple structure for covariance matrices reduces the number of parameters to be estimated. It gives the ability both to assign absence data to a single cluster of habitat condition and to associate some measure of uncertainty to this assignment as probabilities.

Among the different geographic, trophic competition, and fast ice variables, the most contributing variables explaining emperor penguin habitats were fast ice persistence, the slope of the bathymetry, and the magnitude of the fast ice annual cycle. Fast ice variables appear to influence the emperor presence in a different way, depending on the habitat (cluster). Prediction of habitat clusters for absence data using the model-based clustering model built with presence data shows that emperor penguins inhabit regions with various fast ice characteristics: The Weddell Sea region had intermediate fast ice persistence and low magnitude of annual cycle; East Antarctica was dominated by high fast ice persistence and high magnitude, and the Ross Sea was described by low fast ice persistence and low magnitude of the annual cycle. Such regional differences in the direction of an environmental effect on a population/species is not rare [see review by (*43*)], and habitat-species relationships were shown to vary between colonies, even for those hundreds of kilometers apart, for several species. Such examples include, e.g., Scopoli’s shearwaters (*Calonectris Diomedea*) at four colonies in the Mediterranean sea (*44*), Cassin’s auklet (*Ptychoramphus aleuticus*) in the southern and northern California currents (*45*), and snow petrels (*Pagodroma nivea*) at two different colonies in Antarctica (*46*). These studies, complemented by ours, raise the question of whether spatio-temporal extrapolation/transferability in habitat modeling is biologically meaningful, correct, and robust [see review by (*47*)]. For example, intrinsic predictability was shown to be spatially variable across populations of Adélie penguin (*48*). Even by using detailed times series across various colonies, the spatial forecast horizons for Adélie penguin breeding colonies were unexpectedly short.

Regarding the other fast ice variables with a contribution above 20%, most clusters had low median fast ice extent (except cluster 3), low volatility, and fast ice trend close to zero or a tendency to be negative for clusters 1, 3, and 4. These results are in agreement with all previous studies mentioning the need for a stable fast ice platform (i.e., no strongly negative trends and low volatility) and an easy access to foraging areas (i.e., short distances and low but nonzero fast ice extent) for feeding the chicks during the breeding season (*13*, *34*, *49*). Unexpectedly, most clusters had a timing of fast ice minimum later in the season (after March), but we are yet to find a biological reason. One possible explanation would be the existence of a stable platform early in the season for them to start breeding or shorter distance to walk to the colony location upon return in autumn/beginning of winter. Density dependence and trophic competition also differ between areas and may be correlated with local prey availability and/or whether these areas host other competitive species (i.e., Weddell seals or Adélie penguins). Distance to Weddell seals was not an important contributing variable; however, we did not take into account colony size, while in (*50*), they showed a relationship between distances to Weddell seals, specifically, for large colonies of emperor penguins. Colonies with numerous birds were associated with lower presence of Weddell seals. Distance to Adélie penguin colonies appears to be an important contributing variable as we observed that emperor penguin colonies maintain a similar distance from Adélie penguin colonies (between 15 ± 45 km and 64 ± 69 km on average), except for the Weddell Sea region (cluster 1). However, this latter result was expected, as there are few Adélie penguin colonies in this region (*21*, *50*, *51*).

We do not know whether we are characterizing the fundamental niche or the realized ones, and the absence of differences (for most clusters and variables; despite the sample size) between presence and absence data may be associated with this question. Organisms do not always occupy all best suitable habitats (or conversely, they may occupy unsuitable ones), either as a result of dispersal barriers, gregarious behavior, anthropogenic disturbances, biotic exclusion (e.g., competition, parasitism), or simply because these habitats no longer exist (*47*). The emperor penguin presence data at our disposal may not always correspond to the best suitable habitats but rather to a combination of suitable environmental conditions (including the ones not taken into account), presence of physical barriers to access breeding sites such as ice shelves or icebergs, and the ratio between prey availability and intra/inter specific competition for resources.

Here, we reveal the existence of a range of diverse habitats, and the four habitat regions that we identify (Fig. 3A) appear to correspond to four distinct genetic metapopulations identified by (*30*), leading to the conclusion that multiple, regional metapopulations existed rather than a single panmictic population. Cluster 1 corresponds to the Weddell Sea metapopulation, the three colonies associated with cluster 3 correspond to the Mawson coast metapopulation, cluster 5 to the East Antarctic coast metapopulation, and cluster 2 to the Ross Sea metapopulation (black polygons on Fig. 3A). Adaptation/plasticity to specific conditions (e.g., the three colonies on the Mawson coast) may be associated with specific genetic structure; the identified breeding populations and habitats should be considered as separate units for management and populations projections (*30*), as certain metapopulations may be able to survive in certain conditions and others not. We posit that regions with a mixture of different habitat clusters may represent regions where emperor penguin dispersal have mixed the genetic structure [for example, the region from Cape Colbeck (∼157°W) to Smith (∼60°W), which corresponds to a mixture between classes 2, 4, and 5; Fig. 3A]. Several studies have observed emperor penguin shifting colony sites to more favorable places (*52*–*54*). Although emperor penguins are philopatric; they may occasionally disperse massively, which could facilitate gene flow, thus potentially providing new, adaptative alleles (*30*). However, the impact of dispersal on the future global population size is relatively small compared to the impact of climate change mitigation (*7*, *55*).

Our study raises the question of whether certain habitats have a better quality than others and would host larger size colonies than others. Given that our study showed that emperor penguins seem to breed in a wider range of habitats than originally thought, short to medium-term adaptation strategies to habitat change and variability may include splitting into smaller colonies. However, this would assume that there are suitable areas to shift to in the future, which leads to the need for future research to continue monitoring the size and location of emperor penguin colonies to determine their fate. We assessed the spatial sorting of colonies by population size estimated in 2009 (*56*). Two areas where colonies with a larger size than the population average (in red; fig. S7) are located within the area of clusters 1 and 2 (Weddell Sea and Ross Sea regions), while colonies smaller than the average (in blue; fig. S7) are located mainly within the other clusters 3 to 5 (remaining regions). Our research suggests that some habitats may be more favorable than others to host larger colonies; however, further research is needed to clarify these links. Mechanistic linkages between population size and habitats may result from a combination of factors, but we were not able to consider prey availability in our study, which may be an important factor driving emperor penguin habitats, along with the other parameters studied here. We hypothesized that if larger colonies are associated with better habitat quality, then smaller emperor penguin colonies are more vulnerable to impacts and more likely to decrease, and thus that conservation efforts should focus on the areas where the larger colonies reside, as these may be more resilient to impacts. However this remains to be determined.

In terrestrial and marine Antarctic ecological studies, the habitat of a given species is often considered homogeneous, while metapopulations may exist and have different habitat-species relationships (*57*), adaptive (e.g., behavioral plasticity and microevolutionary processes), and dispersal abilities (*58*). This may increase species resilience under climate change/variability (*43*, *59*). For the first time, our study describes a range of diverse habitats for emperor penguins, with different sea ice conditions matching the existing genetic metapopulation (*30*). Given the projection of quasi-extinction of this species due to global warming, this study provides essential information for the conservation of this species on short to medium terms, and is an ongoing work that should be updated regularly for medium-term management.

## MATERIALS AND METHODS

### Experimental design

For the purpose of this work, we only used areas where fast ice is present; to achieve this, a mask was applied so that all pixels with a fast ice persistence below 10^−6^ were removed. The initial grid resolution for fast ice variables was of 0.025° (i.e., ∼1 km). Emperor penguin presence cells were all the cells within 3 km of the 55 emperor penguin colony locations. The grid was then aggregated to a 5-km grid (0.1°) for all variables.

### Fast ice variables

Fast ice variables were derived from the recent publication of a new unique time series of fast ice extent (*18*) at a 1 km and 15-day resolution, generated by compositing cloud-free visible and thermal infrared imagery from NASA MODIS sensors onboard the Terra and Aqua satellites (*19*, *20*). It is important to mention that these are all per-pixel quantities and require no spatial averaging to obtain these quantities. Seven variables were derived from this dataset from March 2010 to March 2018. The time period was selected to match with the ongoing circumpolar satellite monitoring of emperor penguin populations to provide the possibility of combining our findings with population estimates in further research. Volatility (i) is a measure of the short time scale (1 month) variability of fast ice coverage. It is calculated by subtracting from the raw time series a three-point (i.e., ±15 days) boxcar-smoothed time series (three points are about 45 days wide). This provides the “high-frequency” signal of fast ice coverage. Then, for each pixel, we calculated the root mean square of the result, providing a single number for volatility for each pixel between 0 and 1. Persistence (ii) is a simple measure of the mean fast ice residence across the 9 year time series. A 0% indicates that fast ice never covers a pixel, while 100% indicates that the pixel was permanently covered. The timing of fast ice maximum and minimum (iii and iv) represent the time of year when that pixel tends to reach max/min coverage. It is calculated by fitting a fourth-order Fourier series to 13-point (i.e., approximately 6 months) boxcar-smoothed data, then calculating the time of year when that sinusoid reaches minimum/maximum. We then transform the date for fast ice maximum into a categorical value as follows: early was −5 (July), median 0 (September), and late was 5 (December). Values of min and max timing of fast ice are discarded (multiplied by 0) when the magnitude of the annual cycle is below 0.4, because these values represent regions of extremely high or low fast ice persistence, so timing results are noisy/biased. For fast ice minimum, the timing was coded as follows: early was −5 (December), median 0 (March), late was 5 (May). The magnitude of the annual cycle (v) is a measure of the magnitude of the annual cycle of fast ice (values between 0 and 1). The magnitude is zero both in regions of 0 and 100% persistence. As with the calculation of the timing of fast ice maximum and minimum, it is calculated by fitting a Fourier series to a 13-point smoothed time series, then retrieving the magnitude of that series. The fast ice trend (vi) is the trend calculated from March 2010 to 2018 per cell, and the fast ice extent (vii) was calculated from the median of the first 15 days of October among years per cell in kilometer. All Fourier fitting was done using the MPFIT routine implemented in IDL (*60*), which implements the Levenberg-Marquardt algorithm for least squares minimization.

There is a bell-shaped curve relationship between fast ice volatility and persistence and also between the magnitude of the fast ice annual cycle and persistence; for medium persistence there is a high volatility (i.e., forms and breaks on short time scale) and an high amplitude of the magnitude of the fast ice annual cycle (fig. S5).

### Bathymetric and biological variables

Following procedures previously described in (*61*), a bathymetric grid of the Southern Ocean was obtained at a 500 m horizontal resolution [IBCSO v1.0; (*62*)]. Within ArcMap, a land and ice shelf layer (*63*) was used to mask out these areas from the bathymetric grid. From this layer then, a bathymetry line shapefile at −800 m was created using the Contour tool of ArcGIS; slope was calculated in degrees using the Slope tool of ArcGIS. To derive the mean depth and 365 mean slope, the bathymetric grid and slope grid were averaged across 10 × 10 500-m cells using the Aggregate tool of ArcGIS. The Aggregate tool allows for the creation of grids at different resolutions. Because we aggregated by 10 × 10 cells, the resulting grid is of 5 km resolution. The 5-km grids was then used to create the 5-km sampling location shapefile (i.e., the cell centroids from the raster) using the Raster to Point tool of ArcGIS. Each 5-km cell was then associated with a mean slope and bathymetry. We then calculated distance to the 800-m isobath using the Near tool of ArcGIS. The resulting shapefile was then ready for use in the R environment. Within R, each sampling location was further attributed with spatially overlapping grid values or distances to shapefile features. Last, the distances to the nearest Adélie penguin colony were derived from (*64*, *65*) to the nearest Weddell seals from (*66*) and to the nearest emperor penguin colony were computed for each cell of the 5-km grid.

### Statistical analysis

#### Emperor penguin habitat

The dataset contains *M* = 177 cells with emperor penguin presence data compared with *N* = 58,580 cells of absence data within fast ice. We dispose of a presence/absence variable to predict that it would have been preferable to direct the analysis toward some suitable supervised statistical methods such as classification tree, discriminant analysis, or logistic regression. However, we face an important problem of unbalanced dataset (177 presences versus 58,580 absences); even if the model never predicts the presence, we would have about 99.7% chance of a good prediction accuracy when predicting only absence, with no possibility to improve the model. Moreover, presence data were neither heavily associated with a single variable nor a threshold, as shown with distribution between presence and absence data for each variable (fig. S6). We ran a PC analysis on a correlation matrix for presence data (function PCA from R package FactoMineR; step 1 in Fig. 1; please see details in the next section). This implies that same weight is given to the variables in the analysis by scaling their variance to unity. Once the first four PCs were obtained, observations of absence data are projected in their space391 (function predict.PCA from R package FactoMineR), accounting for a sufficient amount of variability (90%). The idea of running a PC analysis on presence data is to shape the habitat space with the environmental conditions of the presence. Projecting the absence onto the structure of the presence’s PC analysis space helps visualize any differences between presence and absence. The environmental conditions of an absence value far from the environmental conditions of presence values will be made more evident with this transformation of the covariate dataset.

### Constructing clusters of habitat

The objectives and design of the study are presented in the workflow (Fig. 1). Start by computing the correlation matrix for presence dataset using *P* variables describing habitat conditions. Step I is a dimension reduction step achieved with PC analysis on the correlation matrix giving rise to a small number *Q* < *P* of composite variables (the PCs). The *M* observations of presence dataset are represented in their *Q* PC space of reduced dimension. Step II provides clusters of habitat conditions with model-based clustering using the *Q* composite variables of presence data as predictors. The Gaussian mixture model gives the ability to assign a probability of belonging to a cluster for each observation. This leads to step III: using the previous clusters of habitat conditions, the *N* absence data are projected in the PC space of presence dataset and are assigned to a class with some probabilities. It is then possible to map these probabilities in the geographical space and to appreciate the vicinity of suitable habitat conditions around the Antarctica continent. Another way to proceed is provided with step IV: Using the joint density (the mixture Gaussian distribution) provided with the model-based clustering, one can compute a measure of closeness to optimal habitat conditions as a probability of belonging to favorable habitat conditions (see details below). Spatial mapping of these quantities can also be realized for the entire set of observations (presence and absence data).

Clusters of habitat are constructed using a Bayesian approach with model-based clustering model-based clustering is an unsupervised method of clustering in which a statistical model is fit to data to identify the inherent groupings. Starting with the sample **x**_1_, …, **x**_**N**_ of the *M* presence data projected in a *Q*-dimensional space of previous PC analysis (*Q* = 4 accounts for more than 55% of explained variance), it is assumed that they are generated from a multivariate density *p*(x) considered as a finite mixture of component models of the form

$$p(x)=\sum_{k=1}^{K}\pi_{k}\phi_{k}(x;{\text{μ}}_{k},{\text{Σ}}_{k})$$

Component ϕ*_k_* is assumed to be a multivariate Gaussian distribution with parameters **μ***_k_* (mean vector) and **Σ***_k_* (covariance matrix). If the number *K* of components is known, as well as the previous coefficients, including mixture coefficients π_1:*K*_, this distribution model for the environmental variables allows to assign a class *z_n_* ∈ {1, ⋯, *K*} to each observation **x***_n_*. One may define a joint distribution over the couples (*z_n_*, **x***_n_*),*m* = 1,…,*M*, and using the Bayes rule, a discrete conditional distribution of *z_n_* is associated to each observation **x***_n_* and is given with

$$p(z_{n}=k\;[|]x_{n})=\frac{p(z_{n}=k)p(x_{n}\;[|]z_{n}=k)}{p(x_{n})}=\frac{\pi_{k}\phi_{k}(x_{n};{\text{μ}}_{k},{\text{Σ}}_{k})}{\sum_{l=1}^{K}\pi_{l}\phi_{l}(x_{n};{\text{μ}}_{l},{\text{Σ}}_{l})},k=1,\cdots ,K$$

These conditionals reflect the updated beliefs concerning *z_n_* after **x***_n_* is observed. Before **x***_n_* is observed, the prior belief that it belongs to cluster *k* has probability π*_k_*. After **x***_n_* is observed, this belief is updated considering the likelihood of **x***_n_* under each Gaussian component. The conditional distribution provides what is called a soft clustering since some probability is assigned to observation **x***_n_* to belong to cluster *k*. If a hard clustering is required, observation **x***_n_* is assigned to a single class *z_n_* by selecting the value of *k* ∈ {1, …, *K*} for which the conditional distribution *p*(*z_n_* = *k* [∣]**x***_n_*) is maximum (maximum likelihood estimation). If *a_n_* = max_*k*=1,…,*K*_{*p*(*z_n_* = *k* [∣]**x***_n_*)} defines a measure of accuracy for the classification of observation **x***_n_*, the quantity *u_n_* = 1 − *a_n_* is a measure of uncertainty of that classification. In the same way, the joint distribution *p*(**x**) may be used for constructing a measure of closeness to optimal habitat conditions defined by high-density regions of this distribution. Suppose an observation **x***_n_* and define the domain 𝒟*_n_* = {**x** ∈ ℝ^4^ s. t. *p*(**x**) ≥ *p*(**x***_n_*)}, which corresponds to the domain (connex or not) enclosed by the contour line of the density with height *p*(**x***_n_*). Compute the probability *p_n_* = ∫_𝒟*_n_*_ ‍ *p*(**x**)d**x**, which goes to 1 as **x***_n_* moves far from the cloud of points. Then, the quantity *c_n_* = 1 − *p_n_* defines a measure of closeness to high-density region of optimal habitat. From a practical point of view, the value *p_n_* is approximated numerically (trapezoidal rule), which may be time-consuming.

The R package mclust is used to estimate the number of components *K* and the parameters **μ**_1:*K*_, **Σ**_1;*K*_, and π_1:*K*_ using a numerical method (expectation-maximization algorithm). It provides some advisable criteria for model selection, such as Bayes information criterion, that allow choosing among different candidate models of covariance structure and number of mixture components. Partitions from clusters 2 to 7 were explored using the covariance structure VEI (see entries from function Mclust), which refers to diagonal covariance matrices with varying volume and equal shape across component models.

Once the parameters of the component models have been estimated on presence data, inference can be done on absence data. Start by projecting absence data in the space of the PCs of presence data to construct observations **x***_m_*, *m* = 1, …, *M* constituted with *Q* = 4 PC coordinates. Absence data can now be assigned to a single cluster from those estimated with presence data. Then, both quantities *a_m_*, the measure of classification accuracy, and *c_m_*, the measure of closeness to optimal habitat, are computed for absence data **x***_m_* and are mapped in the spatial domain. The spatial mapping of these probabilities gives the ability to evaluate coastal areas where penguin habitat is potentially optimal.
