# Supplementary material for: Where to live? Landfast sea ice shapes emperor penguin habitat around Antarctica
Source: Sci Adv. 2023 Sep 27;9(39):eadg8340. doi: 10.1126/sciadv.adg8340 (PMC10530227; doi:10.1126/sciadv.adg8340)
Supplement: Supplementary file 1 — Figs. S1 to S7 Tables S1 to S6 [file sciadv.adg8340_sm.pdf]

Science Advances
AAAS

Supplementary Materials for

Where to live? Landfast sea ice shapes emperor penguin habitat around Antarctica

Sara Labrousse *et al.*

Corresponding author: Sara Labrousse, sara.labrousse@locean.ipsl.fr

Sci. Adv. **9**, eadg8340 (2023)
DOI: 10.1126/sciadv.adg8340

This PDF file includes:

Figs. S1 to S7
Tables S1 to S6

Supplementary materials

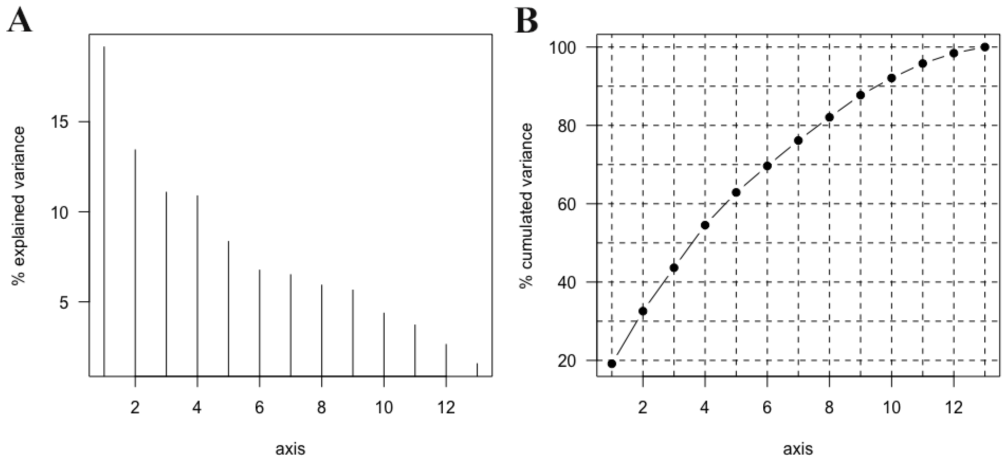

Figure S1: **Representation of the percentage of variance explained by each component (A) and the cumulated variance (B) of the principal component analysis.**

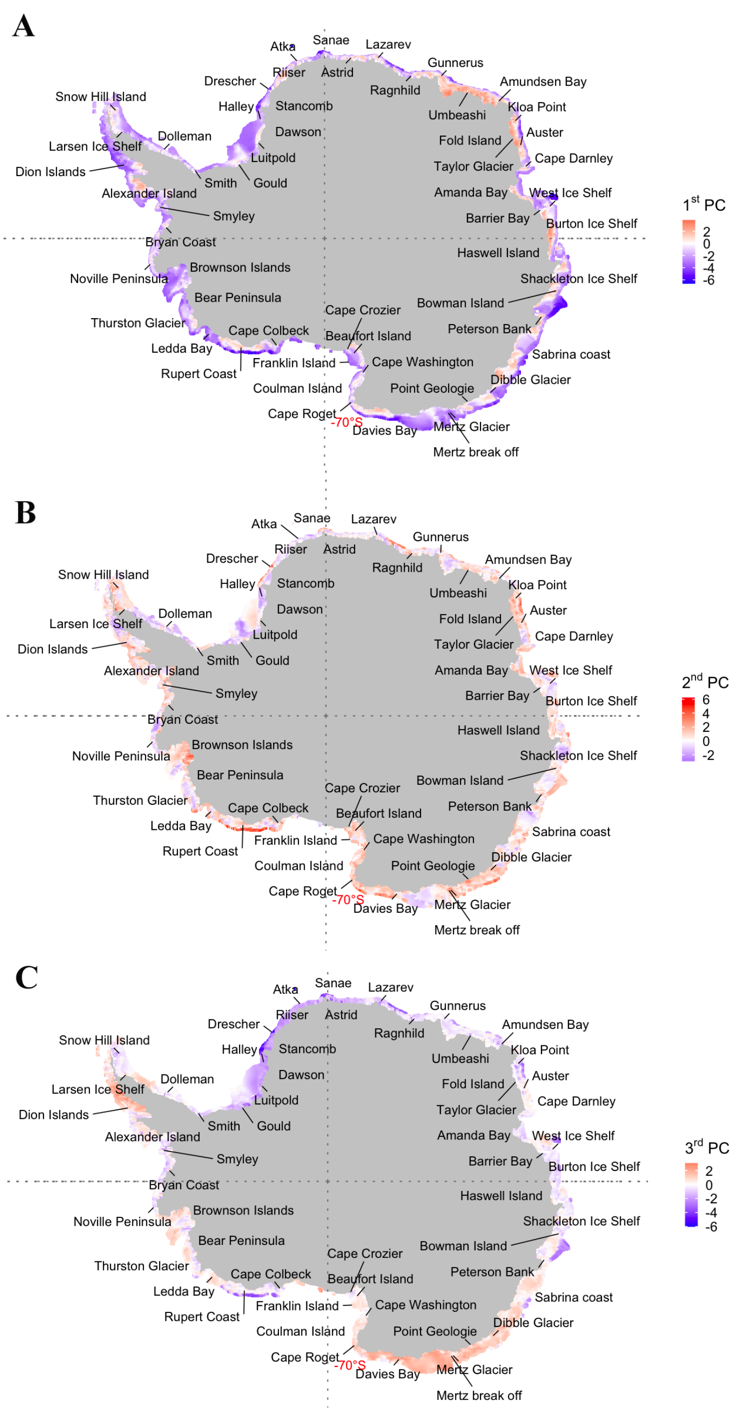
A
Atka
Sanae
Lazarev
Riiser
Astrid
Gunnerus
Drescher
Amundsen Bay
Ragnhild
Snow Hill Island
Halley
Stancomb
Umbeashi
Kloa Point
Dawson
Auster
Dolleman
Larsen Ice Shelf
Luitpold
Fold Island
Cape Darnley
Dion Islands
Smith
Gould
Taylor Glacier
Alexander Island
Amanda Bay
West Ice Shelf
Smyley
Barrier Bay
Burton Ice Shelf
Bryan Coast
Haswell Island
Brownson Islands
Noville Peninsula
Shackleton Ice Shelf
Bear Peninsula
Cape Crozier
Bowman Island
Thurston Glacier
Cape Colbeck
Beaufort Island
Peterson Bank
Ledda Bay
Franklin Island
Cape Washington
Sabrina coast
Rupert Coast
Coulman Island
Point Geologie
Dibble Glacier
Cape Roget
-70°S
Davies Bay
Mertz Glacier
Mertz break off
1st PC
2
0
-2
-4
-6
B
2nd PC
6
4
2
0
-2
C
3rd PC
2
0
-2
-4
-6

Figure S2:

Figure S2: (Previous page) **Spatial mapping of predicted principal components (PCs) of absence data conditionally to the structure of the emperor penguin presence data from the principal component analysis space.** (A), (B) and (C) correspond to the PC1, PC2 and PC3 coordinates respectively. For the first component, 3 main regions emerged (Fig. S2A): 1) $20°$ W (Riiser colony) to $70°$ E (Auster colony) characterized in majority by positive values of fast ice persistence and magnitude of the annual cycle (this means relatively high fast ice persistence and high amplitude in the seasonal cycle); 2) $70°$ E (Auster colony) to $130°$ W (Dibble glacier colony) characterized by a mixture between positive and negative values of fast ice persistence and magnitude; and 3) $130°$ W (Dibble glacier colony) to $20°$ W (Riiser colony) characterized by mainly negative values of fast ice persistence and magnitude of the annual cycle (this means relatively low fast ice persistence and low amplitude in the seasonal cycle). Regarding the second component (which was slope; Fig. S2B), no clear regional pattern is observed. For the third component (distance to Adélie penguin; Fig. S2C), the studied region is split into two regions from $50°$ W (Dolleman colony) to $100°$ E (Bowman Island colony) mainly represented by positive values (this means relatively short distances to Adélie penguin colonies and long distances to the isobath $800m$) and from $100°$ E to $50°$ W represented by negative values of the third component (the contrary).

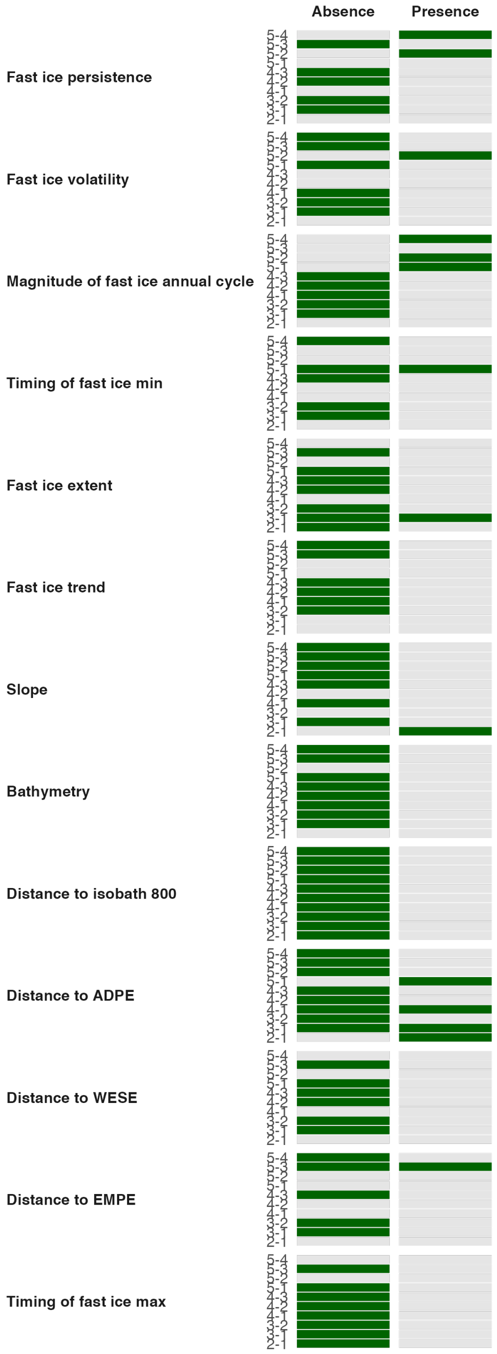

Figure S3: **Significant difference (Wilcoxon test on medians) between habitat clusters for each variables and presence and absence data.** The significance is represented by the green colour for a threshold of 5%. Only data with 80% certainty in the cluster attribution were used for this analysis.

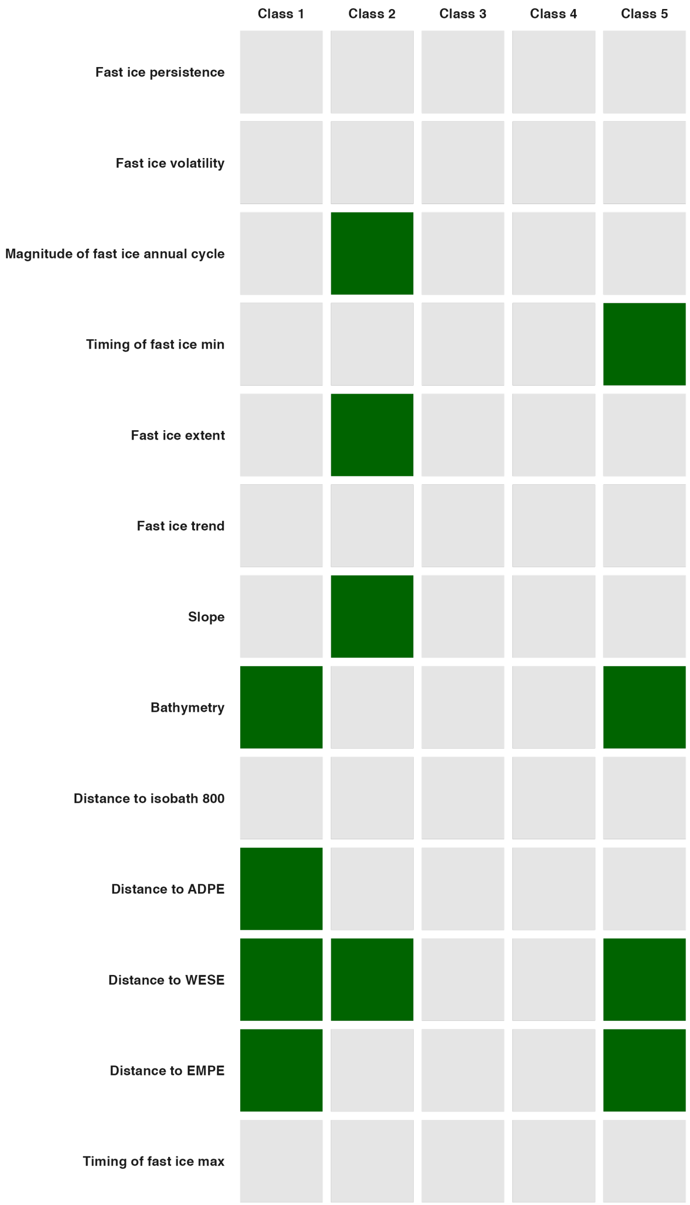

Figure S4: **Significant difference (Wilcoxon test on medians) between presence and absence data for each variables and habitat clusters.** The significance is represented by the green colour for a threshold of 5%. Only data with 80% certainty in the cluster attribution were used for this analysis.

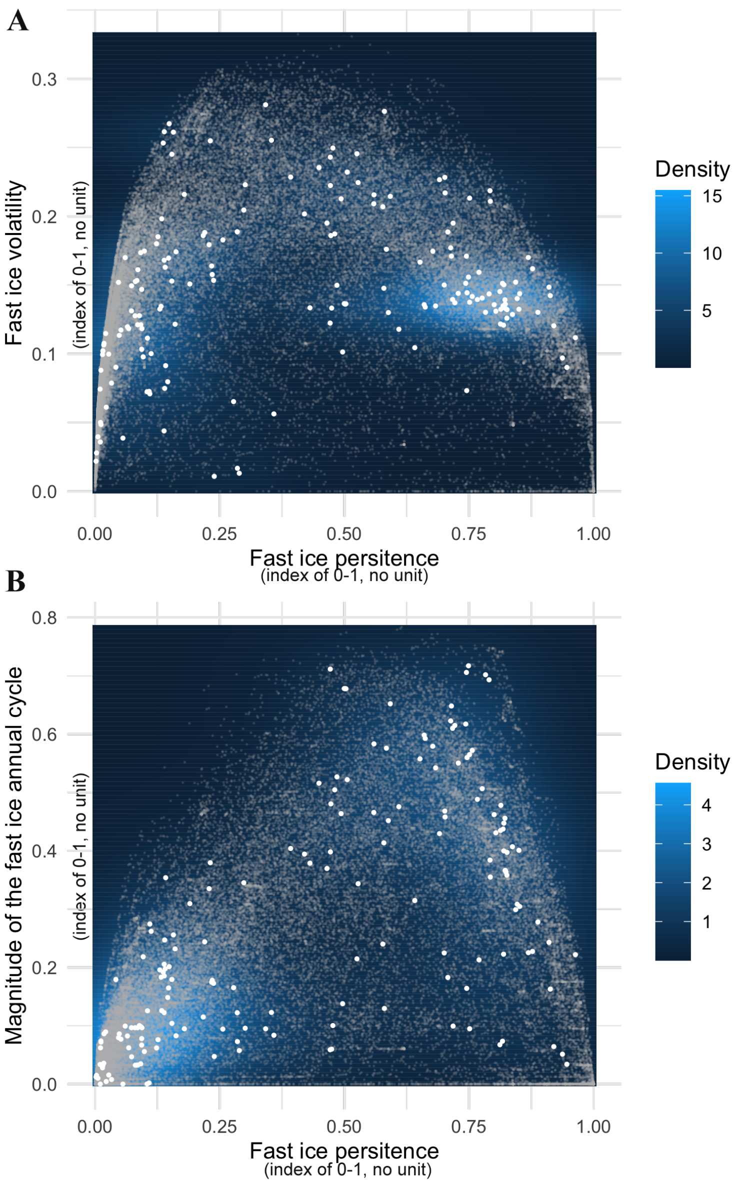

Figure S5: **Scatter plot and density plot representing the relationships between fast ice persistence and fast ice volatility (A) and the magnitude of fast ice annual cycle (B).** Plain white dots represent the presence data while grey small dots represent the absence data. The density distribution of presence data is represented on the background.

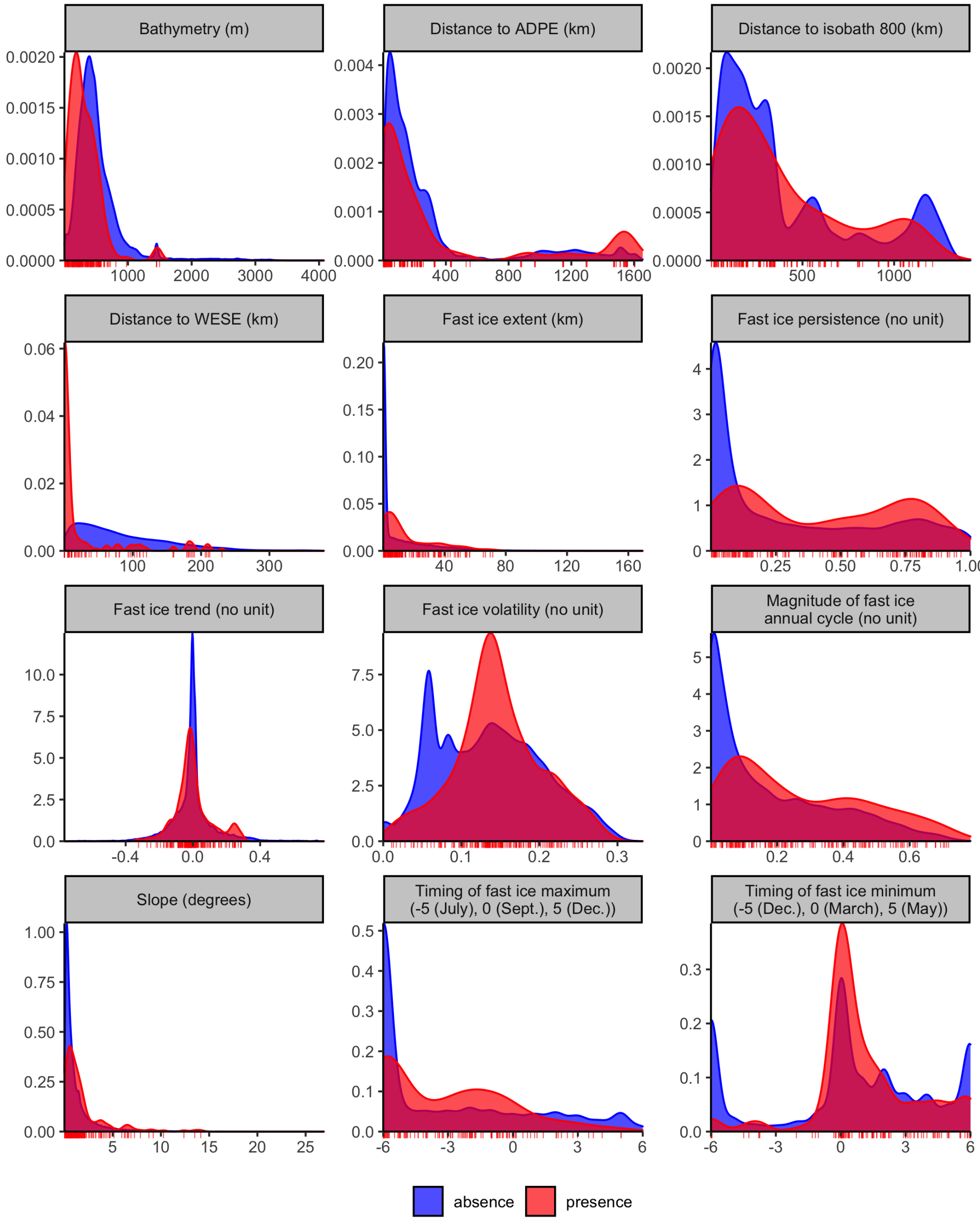

Figure S6: **Density plot distribution for each variable for presence (red colour) and absence data (blue colour).**

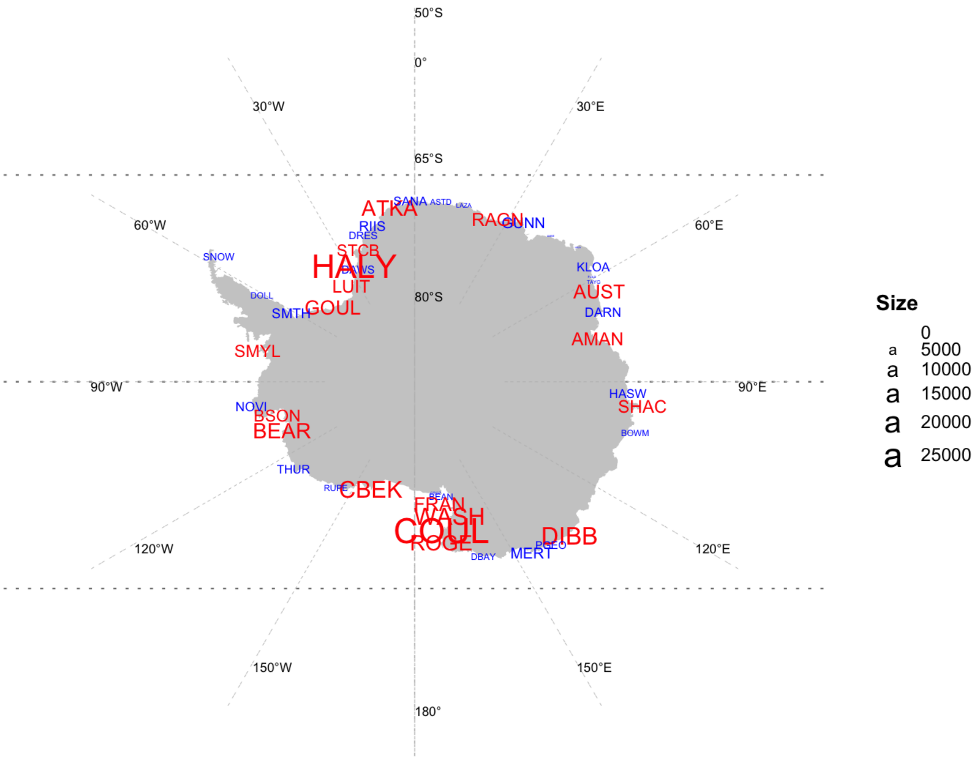

Figure S7: **Geographic representation of colony size from [56]** with higher size than the population average represented in red while colonies with size under the population average are represented in blue. The size of the colony names represents the colony size.

Table S1: **Median and standard deviation values for all variables and all habitat clusters for presence and absence data.** Only data with 80% certainty in the cluster attribution were used for this analysis. Acronyms are defined as follows: Adélie penguin (ADPE), emperor penguin (EMPE), Weddell seal (WESE) colonies.

presence	class	variable	median	std
absence	1	Fast_ice_persistence	0,24	0,29
absence	1	fast_ice_volatility	0,17	0,07
absence	1	Magnitude_of_fast_ice_annual_cycle	0,18	0,14
absence	1	Timing_of_fast_ice_min	3,05	2,50
absence	1	Fast_ice_extent	2,76	16,31
absence	1	Fast_ice_trend	-0,05	0,13
absence	1	Slope	0,45	0,61
absence	1	Bathymetry	371,86	167,29
absence	1	Distance_to_isobath_800	207,07	175,87
absence	1	Distance_to_ADPE	295,59	514,65
absence	1	Distance_to_WESE	44,72	45,18
absence	1	Distance_to_EMPE	105,60	63,35
absence	1	Timing_of_fast_ice_max	-5,03	3,77
absence	2	Fast_ice_persistence	0,03	0,30
absence	2	fast_ice_volatility	0,08	0,06
absence	2	Magnitude_of_fast_ice_annual_cycle	0,03	0,14
absence	2	Timing_of_fast_ice_min	-0,03	4,03
absence	2	Fast_ice_extent	0,47	17,19
absence	2	Fast_ice_trend	0,00	0,12
absence	2	Slope	0,81	2,13
absence	2	Bathymetry	510,59	453,49
absence	2	Distance_to_isobath_800	274,31	375,82
absence	2	Distance_to_ADPE	119,12	399,32
absence	2	Distance_to_WESE	80,62	67,23
absence	2	Distance_to_EMPE	100,40	60,11
absence	2	Timing_of_fast_ice_max	-6,00	3,51
absence	3	Fast_ice_persistence	0,79	0,08
absence	3	fast_ice_volatility	0,14	0,02
absence	3	Magnitude_of_fast_ice_annual_cycle	0,43	0,11
absence	3	Timing_of_fast_ice_min	1,35	1,40
absence	3	Fast_ice_extent	52,99	14,22
absence	3	Fast_ice_trend	-0,06	0,05
absence	3	Slope	0,89	0,83
absence	3	Bathymetry	225,06	129,73
absence	3	Distance_to_isobath_800	124,66	164,54
absence	3	Distance_to_ADPE	26,86	66,96
absence	3	Distance_to_WESE	11,18	12,54
absence	3	Distance_to_EMPE	49,52	52,18
absence	3	Timing_of_fast_ice_max	2,26	2,52

presence	class	variable	median	std
absence	4	Fast_ice_persistence	0,05	0,08
absence	4	fast_ice_volatility	0,15	0,06
absence	4	Magnitude_of_fast_ice_annual_cycle	0,08	0,08
absence	4	Timing_of_fast_ice_min	3,06	2,79
absence	4	Fast_ice_extent	0,42	2,30
absence	4	Fast_ice_trend	-0,01	0,07
absence	4	Slope	0,33	0,92
absence	4	Bathymetry	440,30	215,42
absence	4	Distance_to_isobath_800	319,48	453,43
absence	4	Distance_to_ADPE	201,67	162,31
absence	4	Distance_to_WESE	132,01	71,94
absence	4	Distance_to_EMPE	164,45	71,96
absence	4	Timing_of_fast_ice_max	-6,00	2,96
absence	5	Fast_ice_persistence	0,69	0,19
absence	5	fast_ice_volatility	0,18	0,04
absence	5	Magnitude_of_fast_ice_annual_cycle	0,44	0,15
absence	5	Timing_of_fast_ice_min	0,96	1,70
absence	5	Fast_ice_extent	15,38	15,61
absence	5	Fast_ice_trend	0,01	0,14
absence	5	Slope	0,60	0,83
absence	5	Bathymetry	348,38	187,29
absence	5	Distance_to_isobath_800	225,49	325,22
absence	5	Distance_to_ADPE	111,14	141,64
absence	5	Distance_to_WESE	29,16	39,80
absence	5	Distance_to_EMPE	99,65	70,74
absence	5	Timing_of_fast_ice_max	-1,39	3,73
presence	1	Fast_ice_persistence	0,24	0,31
presence	1	fast_ice_volatility	0,15	0,06
presence	1	Magnitude_of_fast_ice_annual_cycle	0,13	0,11
presence	1	Timing_of_fast_ice_min	1,86	2,00
presence	1	Fast_ice_extent	5,97	12,20
presence	1	Fast_ice_trend	-0,04	0,11
presence	1	Slope	0,59	1,04
presence	1	Bathymetry	248,01	209,72
presence	1	Distance_to_isobath_800	198,62	174,47
presence	1	Distance_to_ADPE	1149,93	568,82
presence	1	Distance_to_WESE	0,00	34,83
presence	1	Distance_to_EMPE	253,81	102,75
presence	1	Timing_of_fast_ice_max	-5,22	3,33

**presence**	**class**	**variable**	**median**	**std**
presence	2	Fast_ice_persistence	0,21	0,29
presence	2	fast_ice_volatility	0,12	0,05
presence	2	Magnitude_of_fast_ice_annual_cycle	0,17	0,18
presence	2	Timing_of_fast_ice_min	0,01	2,57
presence	2	Fast_ice_extent	4,29	17,56
presence	2	Fast_ice_trend	0,04	0,11
presence	2	Slope	3,09	3,95
presence	2	Bathymetry	455,10	354,90
presence	2	Distance_to_isobath_800	603,62	393,25
presence	2	Distance_to_ADPE	32,27	407,79
presence	2	Distance_to_WESE	7,07	37,94
presence	2	Distance_to_EMPE	151,65	117,52
presence	2	Timing_of_fast_ice_max	-2,93	2,80
presence	3	Fast_ice_persistence	0,82	0,07
presence	3	fast_ice_volatility	0,13	0,00
presence	3	Magnitude_of_fast_ice_annual_cycle	0,42	0,07
presence	3	Timing_of_fast_ice_min	1,00	0,40
presence	3	Fast_ice_extent	66,04	9,23
presence	3	Fast_ice_trend	-0,06	0,02
presence	3	Slope	1,86	0,76
presence	3	Bathymetry	126,72	34,82
presence	3	Distance_to_isobath_800	177,47	198,37
presence	3	Distance_to_ADPE	15,64	45,92
presence	3	Distance_to_WESE	2,50	3,21
presence	3	Distance_to_EMPE	66,48	18,54
presence	3	Timing_of_fast_ice_max	-0,37	1,94
presence	4	Fast_ice_persistence	0,05	0,06
presence	4	fast_ice_volatility	0,13	0,08
presence	4	Magnitude_of_fast_ice_annual_cycle	0,05	0,08
presence	4	Timing_of_fast_ice_min	1,73	3,14
presence	4	Fast_ice_extent	1,10	3,03
presence	4	Fast_ice_trend	-0,02	0,08
presence	4	Slope	0,95	1,19
presence	4	Bathymetry	296,77	171,00
presence	4	Distance_to_isobath_800	440,40	236,64
presence	4	Distance_to_ADPE	64,38	69,49
presence	4	Distance_to_WESE	110,75	79,66
presence	4	Distance_to_EMPE	232,96	119,90
presence	4	Timing_of_fast_ice_max	-5,91	1,56

presence	class	variable	median	std
presence	5	Fast_ice_persistence	0,70	0,16
presence	5	fast_ice_volatility	0,17	0,04
presence	5	Magnitude_of_fast_ice_annual_cycle	0,48	0,16
presence	5	Timing_of_fast_ice_min	0,00	1,21
presence	5	Fast_ice_extent	10,91	15,15
presence	5	Fast_ice_trend	-0,01	0,11
presence	5	Slope	0,93	0,96
presence	5	Bathymetry	167,64	176,98
presence	5	Distance_to_isobath_800	173,60	325,56
presence	5	Distance_to_ADPE	43,13	75,25
presence	5	Distance_to_WESE	0,00	8,34
presence	5	Distance_to_EMPE	233,40	70,49
presence	5	Timing_of_fast_ice_max	-2,11	2,45

Table S2: **Significance values (Wilcoxon test on medians) between habitat clusters for each variables and presence and absence data.** The significance threshold is 5%. Only data with 80% certainty in the cluster attribution were used for this analysis. Acronyms are defined as follows: Adélie penguin (ADPE), emperor penguin (EMPE), Weddell seal (WESE) colonies.

variable	presence	pair	value	significance
Slope	absence	2-1	0,0000	FALSE
Slope	absence	3-1	0,0000	TRUE
Slope	absence	3-2	0,7819	FALSE
Slope	absence	4-1	0,0000	TRUE
Slope	absence	4-2	0,0000	FALSE
Slope	absence	4-3	0,0000	TRUE
Slope	absence	5-1	0,0000	TRUE
Slope	absence	5-2	0,0000	TRUE
Slope	absence	5-3	0,0000	TRUE
Slope	absence	5-4	0,0000	TRUE
Bathymetry	absence	2-1	0,0000	FALSE
Bathymetry	absence	3-1	0,0000	TRUE
Bathymetry	absence	3-2	0,0000	TRUE
Bathymetry	absence	4-1	0,0000	TRUE
Bathymetry	absence	4-2	0,0000	TRUE
Bathymetry	absence	4-3	0,0000	TRUE
Bathymetry	absence	5-1	0,0000	TRUE
Bathymetry	absence	5-2	0,0000	FALSE
Bathymetry	absence	5-3	0,0000	TRUE
Bathymetry	absence	5-4	0,0000	TRUE
Distance_to_isobath_800	absence	2-1	0,0000	TRUE
Distance_to_isobath_800	absence	3-1	0,0000	TRUE
Distance_to_isobath_800	absence	3-2	0,0000	TRUE
Distance_to_isobath_800	absence	4-1	0,0000	TRUE
Distance_to_isobath_800	absence	4-2	0,0000	TRUE
Distance_to_isobath_800	absence	4-3	0,0000	TRUE
Distance_to_isobath_800	absence	5-1	0,0000	TRUE
Distance_to_isobath_800	absence	5-2	0,0000	TRUE
Distance_to_isobath_800	absence	5-3	0,0000	TRUE
Distance_to_isobath_800	absence	5-4	0,0000	TRUE
Distance_to_ADPE	absence	2-1	0,0000	FALSE
Distance_to_ADPE	absence	3-1	0,0000	TRUE
Distance_to_ADPE	absence	3-2	0,0000	TRUE
Distance_to_ADPE	absence	4-1	0,0000	TRUE
Distance_to_ADPE	absence	4-2	0,0000	TRUE
Distance_to_ADPE	absence	4-3	0,0000	TRUE
Distance_to_ADPE	absence	5-1	0,0000	FALSE
Distance_to_ADPE	absence	5-2	0,0000	TRUE
Distance_to_ADPE	absence	5-3	0,0000	TRUE
Distance_to_ADPE	absence	5-4	0,0000	TRUE

variable	presence	pair	value	significance
Fast_ice_trend	absence	2-1	0,0000	FALSE
Fast_ice_trend	absence	3-1	1,0000	FALSE
Fast_ice_trend	absence	3-2	0,0000	TRUE
Fast_ice_trend	absence	4-1	0,0000	TRUE
Fast_ice_trend	absence	4-2	0,0000	TRUE
Fast_ice_trend	absence	4-3	0,0000	TRUE
Fast_ice_trend	absence	5-1	0,0000	FALSE
Fast_ice_trend	absence	5-2	1,0000	FALSE
Fast_ice_trend	absence	5-3	0,0000	TRUE
Fast_ice_trend	absence	5-4	0,0000	TRUE
Fast_ice_persistence	absence	2-1	0,0000	FALSE
Fast_ice_persistence	absence	3-1	0,0000	TRUE
Fast_ice_persistence	absence	3-2	0,0000	TRUE
Fast_ice_persistence	absence	4-1	0,0000	FALSE
Fast_ice_persistence	absence	4-2	0,0000	TRUE
Fast_ice_persistence	absence	4-3	0,0000	TRUE
Fast_ice_persistence	absence	5-1	0,0000	FALSE
Fast_ice_persistence	absence	5-2	0,0000	FALSE
Fast_ice_persistence	absence	5-3	0,0000	TRUE
Fast_ice_persistence	absence	5-4	0,0000	FALSE
Timing_of_fast_ice_min	absence	2-1	0,0000	FALSE
Timing_of_fast_ice_min	absence	3-1	0,0000	TRUE
Timing_of_fast_ice_min	absence	3-2	0,0000	TRUE
Timing_of_fast_ice_min	absence	4-1	0,0065	FALSE
Timing_of_fast_ice_min	absence	4-2	0,0000	FALSE
Timing_of_fast_ice_min	absence	4-3	0,0000	TRUE
Timing_of_fast_ice_min	absence	5-1	0,0000	TRUE
Timing_of_fast_ice_min	absence	5-2	0,0000	FALSE
Timing_of_fast_ice_min	absence	5-3	0,0003	FALSE
Timing_of_fast_ice_min	absence	5-4	0,0000	TRUE
Timing_of_fast_ice_max	absence	2-1	0,0000	TRUE
Timing_of_fast_ice_max	absence	3-1	0,0000	TRUE
Timing_of_fast_ice_max	absence	3-2	0,0000	TRUE
Timing_of_fast_ice_max	absence	4-1	0,0000	TRUE
Timing_of_fast_ice_max	absence	4-2	0,0000	TRUE
Timing_of_fast_ice_max	absence	4-3	0,0000	TRUE
Timing_of_fast_ice_max	absence	5-1	0,0000	TRUE
Timing_of_fast_ice_max	absence	5-2	0,0000	FALSE
Timing_of_fast_ice_max	absence	5-3	0,0000	TRUE
Timing_of_fast_ice_max	absence	5-4	0,0000	FALSE

variable	presence	pair	value	significance
fast_ice_volatility	absence	2-1	0,0000	FALSE
fast_ice_volatility	absence	3-1	0,0000	TRUE
fast_ice_volatility	absence	3-2	0,0000	TRUE
fast_ice_volatility	absence	4-1	0,0000	TRUE
fast_ice_volatility	absence	4-2	0,0000	FALSE
fast_ice_volatility	absence	4-3	0,5971	FALSE
fast_ice_volatility	absence	5-1	0,0000	TRUE
fast_ice_volatility	absence	5-2	0,0000	FALSE
fast_ice_volatility	absence	5-3	0,0000	TRUE
fast_ice_volatility	absence	5-4	0,0000	TRUE
Magnitude_of_fast_ice_annual_cycle	absence	2-1	0,0000	FALSE
Magnitude_of_fast_ice_annual_cycle	absence	3-1	0,0000	TRUE
Magnitude_of_fast_ice_annual_cycle	absence	3-2	0,0000	TRUE
Magnitude_of_fast_ice_annual_cycle	absence	4-1	0,0000	TRUE
Magnitude_of_fast_ice_annual_cycle	absence	4-2	0,0000	TRUE
Magnitude_of_fast_ice_annual_cycle	absence	4-3	0,0000	TRUE
Magnitude_of_fast_ice_annual_cycle	absence	5-1	0,0000	FALSE
Magnitude_of_fast_ice_annual_cycle	absence	5-2	0,0000	FALSE
Magnitude_of_fast_ice_annual_cycle	absence	5-3	0,6986	FALSE
Magnitude_of_fast_ice_annual_cycle	absence	5-4	0,0000	FALSE
Distance_to_WESE	absence	2-1	0,0000	FALSE
Distance_to_WESE	absence	3-1	0,0000	TRUE
Distance_to_WESE	absence	3-2	0,0000	TRUE
Distance_to_WESE	absence	4-1	0,0000	FALSE
Distance_to_WESE	absence	4-2	0,0000	TRUE
Distance_to_WESE	absence	4-3	0,0000	TRUE
Distance_to_WESE	absence	5-1	0,0000	TRUE
Distance_to_WESE	absence	5-2	0,0000	FALSE
Distance_to_WESE	absence	5-3	0,0000	TRUE
Distance_to_WESE	absence	5-4	0,0000	FALSE
Fast_ice_extent	absence	2-1	0,0000	TRUE
Fast_ice_extent	absence	3-1	0,0000	TRUE
Fast_ice_extent	absence	3-2	0,0000	TRUE
Fast_ice_extent	absence	4-1	0,0000	FALSE
Fast_ice_extent	absence	4-2	0,0000	TRUE
Fast_ice_extent	absence	4-3	0,0000	TRUE
Fast_ice_extent	absence	5-1	0,0000	TRUE
Fast_ice_extent	absence	5-2	0,0000	FALSE
Fast_ice_extent	absence	5-3	0,0000	TRUE
Fast_ice_extent	absence	5-4	0,0000	FALSE

variable	presence	pair	value	significance
Distance_to_EMPE	absence	2-1	1,0000	FALSE
Distance_to_EMPE	absence	3-1	0,0000	TRUE
Distance_to_EMPE	absence	3-2	0,0000	TRUE
Distance_to_EMPE	absence	4-1	0,0000	FALSE
Distance_to_EMPE	absence	4-2	0,0000	FALSE
Distance_to_EMPE	absence	4-3	0,0000	TRUE
Distance_to_EMPE	absence	5-1	1,0000	FALSE
Distance_to_EMPE	absence	5-2	1,0000	FALSE
Distance_to_EMPE	absence	5-3	0,0000	TRUE
Distance_to_EMPE	absence	5-4	0,0000	TRUE
Slope	presence	2-1	0,0000	TRUE
Slope	presence	3-1	0,0078	FALSE
Slope	presence	3-2	0,4299	FALSE
Slope	presence	4-1	0,2669	FALSE
Slope	presence	4-2	0,0135	FALSE
Slope	presence	4-3	0,2534	FALSE
Slope	presence	5-1	0,2669	FALSE
Slope	presence	5-2	0,0001	FALSE
Slope	presence	5-3	0,1434	FALSE
Slope	presence	5-4	0,7238	FALSE
Bathymetry	presence	2-1	0,0031	FALSE
Bathymetry	presence	3-1	0,0024	FALSE
Bathymetry	presence	3-2	0,0080	FALSE
Bathymetry	presence	4-1	0,8156	FALSE
Bathymetry	presence	4-2	0,1266	FALSE
Bathymetry	presence	4-3	0,0387	FALSE
Bathymetry	presence	5-1	0,1307	FALSE
Bathymetry	presence	5-2	0,0001	FALSE
Bathymetry	presence	5-3	0,6479	FALSE
Bathymetry	presence	5-4	0,4382	FALSE
Distance_to_isobath_800	presence	2-1	0,0003	FALSE
Distance_to_isobath_800	presence	3-1	1,0000	FALSE
Distance_to_isobath_800	presence	3-2	0,2527	FALSE
Distance_to_isobath_800	presence	4-1	0,0095	FALSE
Distance_to_isobath_800	presence	4-2	1,0000	FALSE
Distance_to_isobath_800	presence	4-3	0,8645	FALSE
Distance_to_isobath_800	presence	5-1	1,0000	FALSE
Distance_to_isobath_800	presence	5-2	0,0025	FALSE
Distance_to_isobath_800	presence	5-3	1,0000	FALSE
Distance_to_isobath_800	presence	5-4	0,0816	FALSE

variable	presence	pair	value	significance
Distance_to_ADPE	presence	2-1	0,0000	TRUE
Distance_to_ADPE	presence	3-1	0,0000	TRUE
Distance_to_ADPE	presence	3-2	1,0000	FALSE
Distance_to_ADPE	presence	4-1	0,0000	TRUE
Distance_to_ADPE	presence	4-2	1,0000	FALSE
Distance_to_ADPE	presence	4-3	1,0000	FALSE
Distance_to_ADPE	presence	5-1	0,0000	TRUE
Distance_to_ADPE	presence	5-2	1,0000	FALSE
Distance_to_ADPE	presence	5-3	0,6530	FALSE
Distance_to_ADPE	presence	5-4	1,0000	FALSE
Fast_ice_trend	presence	2-1	0,0005	FALSE
Fast_ice_trend	presence	3-1	0,9885	FALSE
Fast_ice_trend	presence	3-2	0,0077	FALSE
Fast_ice_trend	presence	4-1	0,9885	FALSE
Fast_ice_trend	presence	4-2	0,0968	FALSE
Fast_ice_trend	presence	4-3	0,3030	FALSE
Fast_ice_trend	presence	5-1	0,1198	FALSE
Fast_ice_trend	presence	5-2	0,1862	FALSE
Fast_ice_trend	presence	5-3	0,0951	FALSE
Fast_ice_trend	presence	5-4	0,7365	FALSE
Fast_ice_persistence	presence	2-1	0,1841	FALSE
Fast_ice_persistence	presence	3-1	0,0169	FALSE
Fast_ice_persistence	presence	3-2	0,0004	FALSE
Fast_ice_persistence	presence	4-1	0,0000	FALSE
Fast_ice_persistence	presence	4-2	0,0017	FALSE
Fast_ice_persistence	presence	4-3	0,0003	FALSE
Fast_ice_persistence	presence	5-1	0,0003	FALSE
Fast_ice_persistence	presence	5-2	0,0000	TRUE
Fast_ice_persistence	presence	5-3	0,0687	FALSE
Fast_ice_persistence	presence	5-4	0,0000	TRUE
Timing_of_fast_ice_min	presence	2-1	0,0001	FALSE
Timing_of_fast_ice_min	presence	3-1	1,0000	FALSE
Timing_of_fast_ice_min	presence	3-2	0,7000	FALSE
Timing_of_fast_ice_min	presence	4-1	1,0000	FALSE
Timing_of_fast_ice_min	presence	4-2	0,7997	FALSE
Timing_of_fast_ice_min	presence	4-3	1,0000	FALSE
Timing_of_fast_ice_min	presence	5-1	0,0000	TRUE
Timing_of_fast_ice_min	presence	5-2	1,0000	FALSE
Timing_of_fast_ice_min	presence	5-3	0,0131	FALSE
Timing_of_fast_ice_min	presence	5-4	0,7997	FALSE

variable	presence	pair	value	significance
Timing_of_fast_ice_max	presence	2-1	0,5848	FALSE
Timing_of_fast_ice_max	presence	3-1	0,0597	FALSE
Timing_of_fast_ice_max	presence	3-2	0,0626	FALSE
Timing_of_fast_ice_max	presence	4-1	0,5848	FALSE
Timing_of_fast_ice_max	presence	4-2	0,0626	FALSE
Timing_of_fast_ice_max	presence	4-3	0,0056	FALSE
Timing_of_fast_ice_max	presence	5-1	0,5848	FALSE
Timing_of_fast_ice_max	presence	5-2	0,8376	FALSE
Timing_of_fast_ice_max	presence	5-3	0,0662	FALSE
Timing_of_fast_ice_max	presence	5-4	0,0565	FALSE
fast_ice_volatility	presence	2-1	0,0059	FALSE
fast_ice_volatility	presence	3-1	1,0000	FALSE
fast_ice_volatility	presence	3-2	1,0000	FALSE
fast_ice_volatility	presence	4-1	1,0000	FALSE
fast_ice_volatility	presence	4-2	0,8263	FALSE
fast_ice_volatility	presence	4-3	1,0000	FALSE
fast_ice_volatility	presence	5-1	0,2979	FALSE
fast_ice_volatility	presence	5-2	0,0000	TRUE
fast_ice_volatility	presence	5-3	0,0059	FALSE
fast_ice_volatility	presence	5-4	0,4434	FALSE
Magnitude_of_fast_ice_annual_cycle	presence	2-1	1,0000	FALSE
Magnitude_of_fast_ice_annual_cycle	presence	3-1	0,0003	FALSE
Magnitude_of_fast_ice_annual_cycle	presence	3-2	0,0266	FALSE
Magnitude_of_fast_ice_annual_cycle	presence	4-1	0,0030	FALSE
Magnitude_of_fast_ice_annual_cycle	presence	4-2	0,0377	FALSE
Magnitude_of_fast_ice_annual_cycle	presence	4-3	0,0003	FALSE
Magnitude_of_fast_ice_annual_cycle	presence	5-1	0,0000	TRUE
Magnitude_of_fast_ice_annual_cycle	presence	5-2	0,0000	TRUE
Magnitude_of_fast_ice_annual_cycle	presence	5-3	1,0000	FALSE
Magnitude_of_fast_ice_annual_cycle	presence	5-4	0,0000	TRUE
Distance_to_WESE	presence	2-1	0,0026	FALSE
Distance_to_WESE	presence	3-1	1,0000	FALSE
Distance_to_WESE	presence	3-2	0,2681	FALSE
Distance_to_WESE	presence	4-1	0,0008	FALSE
Distance_to_WESE	presence	4-2	0,0224	FALSE
Distance_to_WESE	presence	4-3	0,0961	FALSE
Distance_to_WESE	presence	5-1	1,0000	FALSE
Distance_to_WESE	presence	5-2	0,0055	FALSE
Distance_to_WESE	presence	5-3	1,0000	FALSE
Distance_to_WESE	presence	5-4	0,0008	FALSE

variable	presence	pair	value	significance
Fast_ice_extent	presence	2-1	0,6388	FALSE
Fast_ice_extent	presence	3-1	0,0000	TRUE
Fast_ice_extent	presence	3-2	0,0001	FALSE
Fast_ice_extent	presence	4-1	0,0104	FALSE
Fast_ice_extent	presence	4-2	0,0608	FALSE
Fast_ice_extent	presence	4-3	0,0003	FALSE
Fast_ice_extent	presence	5-1	0,0222	FALSE
Fast_ice_extent	presence	5-2	0,0496	FALSE
Fast_ice_extent	presence	5-3	0,0000	FALSE
Fast_ice_extent	presence	5-4	0,0001	FALSE
Distance_to_EMPE	presence	2-1	0,0035	FALSE
Distance_to_EMPE	presence	3-1	0,0132	FALSE
Distance_to_EMPE	presence	3-2	0,3814	FALSE
Distance_to_EMPE	presence	4-1	1,0000	FALSE
Distance_to_EMPE	presence	4-2	0,0785	FALSE
Distance_to_EMPE	presence	4-3	0,0019	FALSE
Distance_to_EMPE	presence	5-1	1,0000	FALSE
Distance_to_EMPE	presence	5-2	0,0045	FALSE
Distance_to_EMPE	presence	5-3	0,0000	TRUE
Distance_to_EMPE	presence	5-4	1,0000	FALSE

Table S3: **Significance values (Wilcoxon test on medians) between presence and absence data for each variables and cluster of habitat.** The significance threshold is 5%. Only data with 80% certainty in the cluster attribution were used for this analysis. Acronyms are defined as follows: Adélie penguin (ADPE), emperor penguin (EMPE), Weddell seal (WESE) colonies.

**variable**	**class**	**pair**	**value**	**significance**
Slope	1	presence-absence	0,1086	FALSE
Bathymetry	1	presence-absence	0,0000	TRUE
Distance_to_isobath_800	1	presence-absence	0,6812	FALSE
Distance_to_ADPE	1	presence-absence	0,0000	TRUE
Fast_ice_trend	1	presence-absence	0,1434	FALSE
Fast_ice_persistence	1	presence-absence	0,2794	FALSE
Timing_of_fast_ice_min	1	presence-absence	0,1599	FALSE
Timing_of_fast_ice_max	1	presence-absence	0,9435	FALSE
fast_ice_volatility	1	presence-absence	0,0809	FALSE
Magnitude_of_fast_ice_annual_cycle	1	presence-absence	0,2925	FALSE
Distance_to_WESE	1	presence-absence	0,0000	TRUE
Fast_ice_extent	1	presence-absence	0,0664	FALSE
Distance_to_EMPE	1	presence-absence	0,0000	TRUE
Slope	2	presence-absence	0,0000	TRUE
Bathymetry	2	presence-absence	0,1012	FALSE
Distance_to_isobath_800	2	presence-absence	0,0135	FALSE
Distance_to_ADPE	2	presence-absence	0,0001	FALSE
Fast_ice_trend	2	presence-absence	0,0911	FALSE
Fast_ice_persistence	2	presence-absence	0,0000	FALSE
Timing_of_fast_ice_min	2	presence-absence	0,0258	FALSE
Timing_of_fast_ice_max	2	presence-absence	0,0058	FALSE
fast_ice_volatility	2	presence-absence	0,1568	FALSE
Magnitude_of_fast_ice_annual_cycle	2	presence-absence	0,0000	TRUE
Distance_to_WESE	2	presence-absence	0,0000	TRUE
Fast_ice_extent	2	presence-absence	0,0000	TRUE
Distance_to_EMPE	2	presence-absence	0,1037	FALSE
Slope	3	presence-absence	0,0127	FALSE
Bathymetry	3	presence-absence	0,0303	FALSE
Distance_to_isobath_800	3	presence-absence	0,1809	FALSE
Distance_to_ADPE	3	presence-absence	0,3636	FALSE
Fast_ice_trend	3	presence-absence	0,6580	FALSE
Fast_ice_persistence	3	presence-absence	0,5425	FALSE
Timing_of_fast_ice_min	3	presence-absence	0,6474	FALSE
Timing_of_fast_ice_max	3	presence-absence	0,0484	FALSE
fast_ice_volatility	3	presence-absence	0,0677	FALSE
Magnitude_of_fast_ice_annual_cycle	3	presence-absence	0,8705	FALSE
Distance_to_WESE	3	presence-absence	0,0136	FALSE
Fast_ice_extent	3	presence-absence	0,0565	FALSE
Distance_to_EMPE	3	presence-absence	0,0592	FALSE

variable	class	pair	value	significance
Slope	4	presence-absence	0,0012	FALSE
Bathymetry	4	presence-absence	0,0107	FALSE
Distance_to_isobath_800	4	presence-absence	0,8048	FALSE
Distance_to_ADPE	4	presence-absence	0,0001	FALSE
Fast_ice_trend	4	presence-absence	0,4877	FALSE
Fast_ice_persistence	4	presence-absence	0,5029	FALSE
Timing_of_fast_ice_min	4	presence-absence	0,1378	FALSE
Timing_of_fast_ice_max	4	presence-absence	0,6994	FALSE
fast_ice_volatility	4	presence-absence	0,7253	FALSE
Magnitude_of_fast_ice_annual_cycle	4	presence-absence	0,3438	FALSE
Distance_to_WESE	4	presence-absence	0,2543	FALSE
Fast_ice_extent	4	presence-absence	0,0029	FALSE
Distance_to_EMPE	4	presence-absence	0,0114	FALSE
Slope	5	presence-absence	0,0447	FALSE
Bathymetry	5	presence-absence	0,0000	TRUE
Distance_to_isobath_800	5	presence-absence	0,0547	FALSE
Distance_to_ADPE	5	presence-absence	0,0001	FALSE
Fast_ice_trend	5	presence-absence	0,4586	FALSE
Fast_ice_persistence	5	presence-absence	0,7175	FALSE
Timing_of_fast_ice_min	5	presence-absence	0,0000	TRUE
Timing_of_fast_ice_max	5	presence-absence	0,0137	FALSE
fast_ice_volatility	5	presence-absence	0,4751	FALSE
Magnitude_of_fast_ice_annual_cycle	5	presence-absence	0,1107	FALSE
Distance_to_WESE	5	presence-absence	0,0000	TRUE
Fast_ice_extent	5	presence-absence	0,9962	FALSE
Distance_to_EMPE	5	presence-absence	0,0000	TRUE

Table S4: **Significance values (Wilcoxon test on medians) between presence and absence data for each variables and cluster of habitat (with the absence data removed within a 10 km buffer).** The significance threshold is 5%. Only data with 80% certainty in the cluster attribution were used for this analysis. Acronyms are defined as follows: Adélie penguin (ADPE), emperor penguin (EMPE), Weddell seal (WESE) colonies.

**variable**	**class**	**pair**	**value**	**significance**
Slope	1	presence-absence	0,1071	FALSE
Bathymetry	1	presence-absence	0,0000	TRUE
Distance_to_isobath_800	1	presence-absence	0,6778	FALSE
Distance_to_ADPE	1	presence-absence	0,0000	TRUE
Fast_ice_trend	1	presence-absence	0,1401	FALSE
Fast_ice_persistence	1	presence-absence	0,2791	FALSE
Timing_of_fast_ice_min	1	presence-absence	0,1585	FALSE
Timing_of_fast_ice_max	1	presence-absence	0,9333	FALSE
fast_ice_volatility	1	presence-absence	0,0767	FALSE
Magnitude_of_fast_ice_annual_cycle	1	presence-absence	0,2855	FALSE
Distance_to_WESE	1	presence-absence	0,0000	TRUE
Fast_ice_extent	1	presence-absence	0,0643	FALSE
Slope	2	presence-absence	0,0000	TRUE
Bathymetry	2	presence-absence	0,0993	FALSE
Distance_to_isobath_800	2	presence-absence	0,0133	FALSE
Distance_to_ADPE	2	presence-absence	0,0001	FALSE
Fast_ice_trend	2	presence-absence	0,0900	FALSE
Fast_ice_persistence	2	presence-absence	0,0000	FALSE
Timing_of_fast_ice_min	2	presence-absence	0,0257	FALSE
Timing_of_fast_ice_max	2	presence-absence	0,0057	FALSE
fast_ice_volatility	2	presence-absence	0,1525	FALSE
Magnitude_of_fast_ice_annual_cycle	2	presence-absence	0,0000	TRUE
Distance_to_WESE	2	presence-absence	0,0000	TRUE
Fast_ice_extent	2	presence-absence	0,0000	TRUE
Slope	3	presence-absence	0,0114	FALSE
Bathymetry	3	presence-absence	0,0236	FALSE
Distance_to_isobath_800	3	presence-absence	0,1761	FALSE
Distance_to_ADPE	3	presence-absence	0,3428	FALSE
Fast_ice_trend	3	presence-absence	0,6340	FALSE
Fast_ice_persistence	3	presence-absence	0,5130	FALSE
Timing_of_fast_ice_min	3	presence-absence	0,6282	FALSE
Timing_of_fast_ice_max	3	presence-absence	0,0423	FALSE
fast_ice_volatility	3	presence-absence	0,0610	FALSE
Magnitude_of_fast_ice_annual_cycle	3	presence-absence	0,8883	FALSE
Distance_to_WESE	3	presence-absence	0,0106	FALSE
Fast_ice_extent	3	presence-absence	0,0547	FALSE

**variable**	**class**	**pair**	**value**	**significance**
Slope	4	presence-absence	0,0011	FALSE
Bathymetry	4	presence-absence	0,0107	FALSE
Distance_to_isobath_800	4	presence-absence	0,8074	FALSE
Distance_to_ADPE	4	presence-absence	0,0001	FALSE
Fast_ice_trend	4	presence-absence	0,4830	FALSE
Fast_ice_persistence	4	presence-absence	0,5077	FALSE
Timing_of_fast_ice_min	4	presence-absence	0,1358	FALSE
Timing_of_fast_ice_max	4	presence-absence	0,7033	FALSE
fast_ice_volatility	4	presence-absence	0,7327	FALSE
Magnitude_of_fast_ice_annual_cycle	4	presence-absence	0,3457	FALSE
Distance_to_WESE	4	presence-absence	0,2567	FALSE
Fast_ice_extent	4	presence-absence	0,0028	FALSE
Slope	5	presence-absence	0,0430	FALSE
Bathymetry	5	presence-absence	0,0000	TRUE
Distance_to_isobath_800	5	presence-absence	0,0511	FALSE
Distance_to_ADPE	5	presence-absence	0,0001	FALSE
Fast_ice_trend	5	presence-absence	0,4590	FALSE
Fast_ice_persistence	5	presence-absence	0,7142	FALSE
Timing_of_fast_ice_min	5	presence-absence	0,0000	TRUE
Timing_of_fast_ice_max	5	presence-absence	0,0130	FALSE
fast_ice_volatility	5	presence-absence	0,4768	FALSE
Magnitude_of_fast_ice_annual_cycle	5	presence-absence	0,1099	FALSE
Distance_to_WESE	5	presence-absence	0,0000	TRUE
Fast_ice_extent	5	presence-absence	0,9959	FALSE

Table S5: **Significance values (Wilcoxon test on medians) between presence and absence data for each variables and cluster of habitat (with the absence data removed within a 20 km buffer).** The significance threshold is 5%. Only data with 80% certainty in the cluster attribution were used for this analysis. Acronyms are defined as follows: Adélie penguin (ADPE), emperor penguin (EMPE), Weddell seal (WESE) colonies.

**variable**	**class**	**pair**	**value**	**significance**
Slope	1	presence-absence	0,0971	FALSE
Bathymetry	1	presence-absence	0,0000	TRUE
Distance_to_isobath_800	1	presence-absence	0,6611	FALSE
Distance_to_ADPE	1	presence-absence	0,0000	TRUE
Fast_ice_trend	1	presence-absence	0,1326	FALSE
Fast_ice_persistence	1	presence-absence	0,2676	FALSE
Timing_of_fast_ice_min	1	presence-absence	0,1397	FALSE
Timing_of_fast_ice_max	1	presence-absence	0,9192	FALSE
fast_ice_volatility	1	presence-absence	0,0649	FALSE
Magnitude_of_fast_ice_annual_cycle	1	presence-absence	0,2783	FALSE
Distance_to_WESE	1	presence-absence	0,0000	TRUE
Fast_ice_extent	1	presence-absence	0,0647	FALSE
Slope	2	presence-absence	0,0000	TRUE
Bathymetry	2	presence-absence	0,0900	FALSE
Distance_to_isobath_800	2	presence-absence	0,0126	FALSE
Distance_to_ADPE	2	presence-absence	0,0001	FALSE
Fast_ice_trend	2	presence-absence	0,0903	FALSE
Fast_ice_persistence	2	presence-absence	0,0000	FALSE
Timing_of_fast_ice_min	2	presence-absence	0,0242	FALSE
Timing_of_fast_ice_max	2	presence-absence	0,0050	FALSE
fast_ice_volatility	2	presence-absence	0,1417	FALSE
Magnitude_of_fast_ice_annual_cycle	2	presence-absence	0,0000	TRUE
Distance_to_WESE	2	presence-absence	0,0000	TRUE
Fast_ice_extent	2	presence-absence	0,0000	TRUE
Slope	3	presence-absence	0,0139	FALSE
Bathymetry	3	presence-absence	0,0098	FALSE
Distance_to_isobath_800	3	presence-absence	0,1460	FALSE
Distance_to_ADPE	3	presence-absence	0,2197	FALSE
Fast_ice_trend	3	presence-absence	0,7346	FALSE
Fast_ice_persistence	3	presence-absence	0,4904	FALSE
Timing_of_fast_ice_min	3	presence-absence	0,4534	FALSE
Timing_of_fast_ice_max	3	presence-absence	0,0169	FALSE
fast_ice_volatility	3	presence-absence	0,0217	FALSE
Magnitude_of_fast_ice_annual_cycle	3	presence-absence	0,9907	FALSE
Distance_to_WESE	3	presence-absence	0,0091	FALSE
Fast_ice_extent	3	presence-absence	0,0620	FALSE

variable	class	pair	value	significance
Slope	4	presence-absence	0,0011	FALSE
Bathymetry	4	presence-absence	0,0106	FALSE
Distance_to_isobath_800	4	presence-absence	0,8143	FALSE
Distance_to_ADPE	4	presence-absence	0,0001	FALSE
Fast_ice_trend	4	presence-absence	0,4861	FALSE
Fast_ice_persistence	4	presence-absence	0,5149	FALSE
Timing_of_fast_ice_min	4	presence-absence	0,1364	FALSE
Timing_of_fast_ice_max	4	presence-absence	0,7112	FALSE
fast_ice_volatility	4	presence-absence	0,7440	FALSE
Magnitude_of_fast_ice_annual_cycle	4	presence-absence	0,3460	FALSE
Distance_to_WESE	4	presence-absence	0,2614	FALSE
Fast_ice_extent	4	presence-absence	0,0029	FALSE
Slope	5	presence-absence	0,0427	FALSE
Bathymetry	5	presence-absence	0,0000	TRUE
Distance_to_isobath_800	5	presence-absence	0,0377	FALSE
Distance_to_ADPE	5	presence-absence	0,0001	FALSE
Fast_ice_trend	5	presence-absence	0,4455	FALSE
Fast_ice_persistence	5	presence-absence	0,6899	FALSE
Timing_of_fast_ice_min	5	presence-absence	0,0000	TRUE
Timing_of_fast_ice_max	5	presence-absence	0,0107	FALSE
fast_ice_volatility	5	presence-absence	0,4583	FALSE
Magnitude_of_fast_ice_annual_cycle	5	presence-absence	0,1009	FALSE
Distance_to_WESE	5	presence-absence	0,0000	TRUE
Fast_ice_extent	5	presence-absence	0,9920	FALSE

Table S6: **Significance values (Wilcoxon test on medians) between presence and absence data for each variables and cluster of habitat (with the absence data removed within a 30 km buffer).** The significance threshold is 5%. Only data with 80% certainty in the cluster attribution were used for this analysis. Acronyms are defined as follows: Adélie penguin (ADPE), emperor penguin (EMPE), Weddell seal (WESE) colonies.

**variable**	**class**	**pair**	**value**	**significance**
Slope	1	presence-absence	0,0829	FALSE
Bathymetry	1	presence-absence	0,0000	TRUE
Distance_to_isobath_800	1	presence-absence	0,5967	FALSE
Distance_to_ADPE	1	presence-absence	0,0000	TRUE
Fast_ice_trend	1	presence-absence	0,1354	FALSE
Fast_ice_persistence	1	presence-absence	0,2671	FALSE
Timing_of_fast_ice_min	1	presence-absence	0,1087	FALSE
Timing_of_fast_ice_max	1	presence-absence	0,9163	FALSE
fast_ice_volatility	1	presence-absence	0,0514	FALSE
Magnitude_of_fast_ice_annual_cycle	1	presence-absence	0,2761	FALSE
Distance_to_WESE	1	presence-absence	0,0000	TRUE
Fast_ice_extent	1	presence-absence	0,0619	FALSE
Slope	2	presence-absence	0,0000	TRUE
Bathymetry	2	presence-absence	0,0813	FALSE
Distance_to_isobath_800	2	presence-absence	0,0125	FALSE
Distance_to_ADPE	2	presence-absence	0,0001	FALSE
Fast_ice_trend	2	presence-absence	0,0921	FALSE
Fast_ice_persistence	2	presence-absence	0,0000	TRUE
Timing_of_fast_ice_min	2	presence-absence	0,0228	FALSE
Timing_of_fast_ice_max	2	presence-absence	0,0043	FALSE
fast_ice_volatility	2	presence-absence	0,1283	FALSE
Magnitude_of_fast_ice_annual_cycle	2	presence-absence	0,0000	TRUE
Distance_to_WESE	2	presence-absence	0,0000	TRUE
Fast_ice_extent	2	presence-absence	0,0000	TRUE
Slope	3	presence-absence	0,0193	FALSE
Bathymetry	3	presence-absence	0,0070	FALSE
Distance_to_isobath_800	3	presence-absence	0,1203	FALSE
Distance_to_ADPE	3	presence-absence	0,1225	FALSE
Fast_ice_trend	3	presence-absence	0,7786	FALSE
Fast_ice_persistence	3	presence-absence	0,5025	FALSE
Timing_of_fast_ice_min	3	presence-absence	0,3861	FALSE
Timing_of_fast_ice_max	3	presence-absence	0,0114	FALSE
fast_ice_volatility	3	presence-absence	0,0202	FALSE
Magnitude_of_fast_ice_annual_cycle	3	presence-absence	0,9414	FALSE
Distance_to_WESE	3	presence-absence	0,0120	FALSE
Fast_ice_extent	3	presence-absence	0,0599	FALSE

variable	class	pair	value	significance
Slope	4	presence-absence	0,0010	FALSE
Bathymetry	4	presence-absence	0,0103	FALSE
Distance_to_isobath_800	4	presence-absence	0,8230	FALSE
Distance_to_ADPE	4	presence-absence	0,0001	FALSE
Fast_ice_trend	4	presence-absence	0,4967	FALSE
Fast_ice_persistence	4	presence-absence	0,5168	FALSE
Timing_of_fast_ice_min	4	presence-absence	0,1408	FALSE
Timing_of_fast_ice_max	4	presence-absence	0,7276	FALSE
fast_ice_volatility	4	presence-absence	0,7458	FALSE
Magnitude_of_fast_ice_annual_cycle	4	presence-absence	0,3439	FALSE
Distance_to_WESE	4	presence-absence	0,2643	FALSE
Fast_ice_extent	4	presence-absence	0,0029	FALSE
Slope	5	presence-absence	0,0421	FALSE
Bathymetry	5	presence-absence	0,0000	TRUE
Distance_to_isobath_800	5	presence-absence	0,0271	FALSE
Distance_to_ADPE	5	presence-absence	0,0000	FALSE
Fast_ice_trend	5	presence-absence	0,4024	FALSE
Fast_ice_persistence	5	presence-absence	0,6834	FALSE
Timing_of_fast_ice_min	5	presence-absence	0,0000	TRUE
Timing_of_fast_ice_max	5	presence-absence	0,0102	FALSE
fast_ice_volatility	5	presence-absence	0,4277	FALSE
Magnitude_of_fast_ice_annual_cycle	5	presence-absence	0,0890	FALSE
Distance_to_WESE	5	presence-absence	0,0000	TRUE
Fast_ice_extent	5	presence-absence	0,9926	FALSE
